# How Digestive Processes Can Affect the Bioavailability of PCBs Associated with Microplastics: A Modeling Study Supported by Empirical Data

**DOI:** 10.1021/acs.est.3c02129

**Published:** 2023-07-28

**Authors:** Nur Hazimah Mohamed Nor, Zhiyue Niu, Marie Hennebelle, Albert A. Koelmans

**Affiliations:** † Aquatic Ecology and Water Quality Management Group, Wageningen University & Research, P.O. Box 47, 6700 AA Wageningen, The Netherlands; § Food Chemistry Group, Wageningen University & Research, P.O. Box 17, 6700 AA Wageningen, The Netherlands

**Keywords:** microplastics, hydrophobic organic chemicals, gut fluid, digestion, lipids

## Abstract

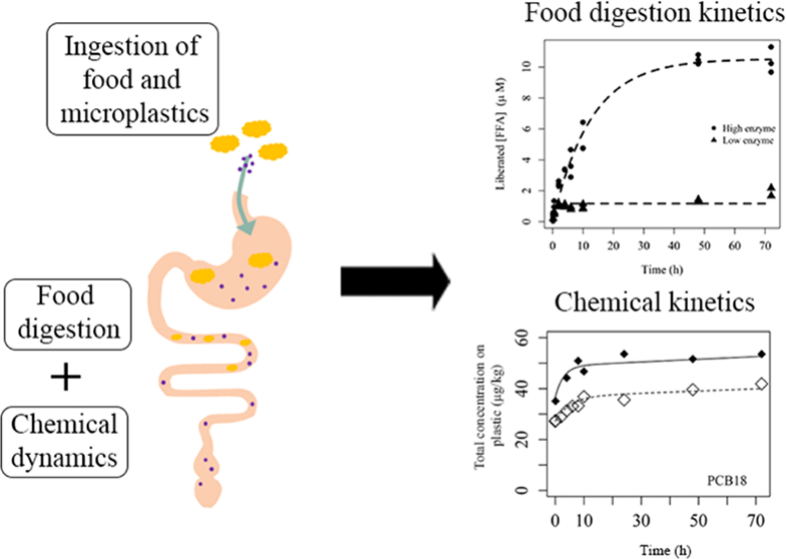

The transfer kinetics of plastic-associated chemicals during intestinal digestive processes is unknown. Here, we assessed whether digestive processes affect chemical exchange kinetics on microplastics, using an *in vitro* gut fluid digestive model mimicking the human upper intestinal tract. Chemical exchange kinetics of microplastics were measured for 10 polychlorinated biphenyls (PCBs) as proxies for the broad class of hydrophobic organic chemicals. Following earlier studies, olive oil was used as a proxy for digestible food, under high and low digestive enzyme activities. The micelle–water and oil–water partition coefficients of the 10 PCBs were also determined to evaluate the relative contribution of each gut component to sorb PCBs. A new biphasic and reversible chemical exchange model, which included the digestion process, fitted well to the empirical data. We demonstrate that the digestive processes that break down contaminated food can lead to a substantial increase in chemical concentration in microplastics by a factor of 10–20, thereby reducing the overall chemical bioavailability in the gastrointestinal tract when compared to a scenario without microplastics. Higher enzyme activities result in more chemicals being released by the digested food, thereby resulting in higher chemical concentrations in the microplastics. While the model-calibrated kinetic parameters are specific to the studied scenario, we argue that the mechanism of the reduced bioavailability of chemicals and the modeling tool developed have generic relevance. These digestive processes should be considered when assessing the risks of microplastics to humans and also biomagnification in aquatic food webs.

## Abstract 2

New biphasic and reversible chemical exchange model with digestion processes to understand the human health effects of microplastics.

## Introduction

The microplastic (MP) vector effect has been widely discussed in the literature and is still one of the major concerns of MP pollution.^1^ When ingested, MPs have the potential to act as vectors and transfer chemicals to organisms under certain conditions.^2^ These plastic particles contain a mixture of chemicals that may either be intentionally or unintentionally added. During the manufacturing process, monomers, plasticizers, and additives are usually added to plastics to achieve the appropriate structure for the designated application.^3,4^ In addition, during the manufacturing process or product conversion, other unintentionally added chemicals may also sorb onto the material.^5^ Another possible source of chemicals in plastics is from ambient water via absorption when the plastics are released into the environment indiscriminately.^6,7^

All biota, including humans, are exposed to MPs (<5 mm).^4^ On the one hand, for animals, plastic consumption can happen when the plastic particles are mistaken for food due to their appearance, or by accidental ingestion due to their feeding method (e.g., filter and deposit feeding), or from the predation of plastic-contaminated organisms.^4^ Based on a collated data for >800 species from 421 studies, approximately 20% of individual aquatic organisms studied so far have plastic or MPs detected in their gastrointestinal tracts (GITs).^8^ On the other hand, for humans, MPs may accidentally be consumed from food and beverages contaminated with MPs. They may also be inhaled from air due to their prevalence in the environment. Human interaction with MPs is inevitable, as we depend on plastic materials in almost every part of our lives (e.g., clothes, food packaging, furniture). Over an average human lifetime of 70 years, it is estimated that an adult can be exposed to up to 20 million particles (1–5000 μm), which is equivalent to 12 mg.^9^ These estimations were based on nine intake media that were known to have MPs and comprise 20% of the human diet. MPs ingested by humans either come from the abrasion and wear-and-tear of plastic products used in our daily lives to process, transport, and store food and beverages or come from bioaccumulation in the food chain (e.g., seafood).^10^

As a carrier for a mixture of chemicals, these plastic particles may release chemicals in the organisms’ digestive tracts when ingested, potentially increasing exposure and risks.^2,11^ An established approach to investigate the chemical vector effect of MPs when ingested is by performing *in vitro* gut fluid experiments. This type of experiment has been widely used to investigate chemical bioavailability from indigestible particles such as sediment, asphalt, and soot in human and animal digestive tracts.^12−16^ Such studies have been increasingly favored to understand the MP vector effect in seabirds, aquatic organisms, and invertebrates for a variety of chemicals.^17−21^ Bioaccessibilities of both organic compounds and heavy metals have also been investigated in different types of *in vitro* setups mimicking the human digestive tract.^22−24^ However, due to the nature of the experiments, these studies can only elucidate the key physicochemical properties of the physiological fluids and polymers that may have affected the chemical bioaccessibility from MPs in each compartment of the digestive tract but not the role of other compartments present, such as food.

Besides measuring chemical bioaccessibility, *in vitro* studies are also useful for estimating chemical exchange kinetics.^20,21,25^ Using chemical kinetic rate constants and other sorption parameters, the chemical behavior has been simulated for different gut retention times to estimate the chemical bioaccessibilities from ingested plastic in different species.^21^ Yet, although the aforementioned study had included food when investigating the chemical exchange in MPs, the food component was an inert pool.^21^ In reality, this is not the case since food is digested, and it is uncertain if the digestion dynamics will affect the chemical exchange kinetics in MPs. It has been well-established that food digestion and absorption in the gastrointestinal tract (GIT) are important factors in chemical uptake from food.^26,27^ However, we are uncertain whether this is the case in the presence of MPs.

When food is digested in the gastrointestinal tract, the composition of the food changes, reducing the chemical fugacity capacities of the GIT content to be below that of the consumed food.^26,27^ Hence, this increases the chemical fugacity of the GIT content above that of the consumed food, which has been demonstrated to be the driving force behind gastrointestinal magnification.^26,27^ We speculate that in the presence of MPs, the chemicals associated with food in the GIT will transfer from the higher fugacity in the food to the lower fugacity in the MPs and other gut constituents.^28−31^ Therefore, MPs may act as a sink and potentially attenuate chemical biomagnification in the biota when the MPs pass through the GIT and are excreted out.^9,21^ To evaluate if MPs act as a major sink for the chemicals and significantly reduce chemical bioaccumulation, the chemical behavior needs to be assessed across all gut components as the food is digested. This includes quantifying the partition coefficients of the chemicals between the other gut components such as food and micelles (which are either produced in the gut or formed from the breakdown of food) and the aqueous phase.

Therefore, in the present study, an environmentally realistic scenario was mimicked, whereby an organism ingests contaminated MPs and contaminated food, followed by food digestion. The aim was to quantify the kinetic rates of chemical transfer to and from microplastics and elucidate the influence of intestinal digestive processes, in this case, lipid digestion as a proxy for food digestion, on the overall chemical bioavailability under simulated gut fluid conditions. To this end, batch setups of simulated gut fluids mimicking the upper human gastrointestinal (GI) tract conditions containing olive oil as food lipids^32^ and low-density polyethylene (LDPE) strips representing ingested MP particles were pre-equilibrated with a range of PCBs to achieve realistic gut chemical conditions. LDPE was chosen here as it is one of the highly produced and used polymer types^33^ and is thus a good proxy for microplastics in general. PCBs were used as model hydrophobic organic compounds (HOCs) because they are commonly found in environmental plastics^34−36^ and in food^37,38^ and can represent the general chemical behavior of HOCs. The chemical dynamics of PCBs in the LDPE were then examined upon lipid digestion as initiated by the addition of lipase under high and low enzyme kinetics, respectively. Polyoxymethylene (POM) passive samplers were used to determine the aquatic phase measurements of PCBs without separating the nonplastic gut fluid components.^39−41^ A previously well-accepted dynamic multicompartment model was redesigned to include the digestion process.^21^ The chemical kinetic parameters for HOCs on MPs during food digestion are discussed in the context of chemical bioavailability and rigorous risk assessments of plastic-associated chemicals for humans and other physiologically similar biota.^9^

## Materials and Methods

### Materials

All chemicals used were of analytical grade or higher purity, and solvents were high-performance liquid chromatography (HPLC) grade. Twelve PCB congeners (IUPAC nos. 18, 28, 52, 77, 101, 118, 138, 153, 156, 169, 180, and 209) with log octanol–water partition coefficients (*K*_OW_)^42^ ranging from 5.24 to 8.18 were prepared in individual stock solutions in either acetone or isooctane at ∼20–100 mg/L. The concentrations of each PCB congener in the spike mixture and the final experimental setup are provided in the Supporting Information (SI) in Table S1. LDPE strips with a thickness of 30 μm were cut from zipper bags found in the supermarket, which were also used for our previous experiment.^21^ They were cut to a final size of 5.1 × 5.1 cm. As the thickness of the strips was 30 μm, this represents the intrapolymer diffusion path lengths similar to those of 30 μm spherical or irregularly shaped environmentally relevant microplastics.^21^ POM strips of thickness 76 μm were obtained from CS Hyde Co. (Lake Villa, IL) and cut into strips with a size of 3.2 × 3.2 cm to be used as equilibrium passive samplers to assess the aqueous phase PCB concentrations after 28 days.^41,43^ Both LDPE and POM strips were cleaned in methanol (MeOH) for 24 h under constant shaking on a horizontal shaker at 150 rpm and then cleaned with Milli-Q water for another 24 h to remove any residual MeOH and air-died before use. Olive oil was purchased from the supermarket (Extra virgin, Carbonell, Spain). Reagents used for preparing the simulated digestive fluids include sodium taurocholate (NaTC) (97%, Alfa Aesar), bovine serum albumin (BSA) (fraction V, ≥96%, Sigma-Aldrich), sodium chloride (Merck Millipore), and sodium azide (99%, Sigma-Aldrich). Lipase FE-01 (>18 000 U/mL) was obtained from ASA Specialenzyme GmbH, Germany.

### Experimental Design

#### Simulated Gut Fluid Digestion Assay

The experiment was carried out sequentially in two phases, whereby the systems containing LDPE, POM, and olive oil in the simulated gut fluid were first shaken for 28 days with PCBs followed by the addition of lipase (time = 0 h), which initiated lipid digestion. Three treatments were set up to simulate high (lipase in its original form) and low (heat-inactivated lipase) enzyme activities and a control treatment to account for any background PCBs from gut fluid components (heat-inactivated lipase with no PCB spike). The high and low enzyme treatments are to compare between high and low metabolic digestive systems, respectively, since all humans have different metabolic rates.

All experiments were performed in 100 mL transparent glass bottles with glass stoppers in quadruplicates. The simulated gut fluid mixture was prepared to achieve final (i.e., second phase of the experiment) concentrations of 10 mM of NaTC and 5 g/L of BSA, constituted in 150 mM sodium chloride solution.^44,45^ Olive oil (7 g), 10 LDPE strips (0.7 g total weight), and a POM passive sampler (0.11 g) were added to each bottle filled with ∼69.9 mL of gut fluid. Note that the mass of plastic used in this experiment may or may not represent the mass of plastic that humans eat daily. This does not disqualify the results, as the targeted kinetic parameters are independent of the mass of plastic. Furthermore, a certain minimum mass of plastic is necessary to meet the chemical analytical minimum detection limits. Following previous studies, to limit biodegradation of the olive oil, sodium azide was added to a final concentration of 25 mg/L.^39,46^ This is 0.002 wt % of the aqueous phase, which is lower than the common range (0.02–0.04%) for inhibiting microbial growth in enzyme assays and thus should not affect the lipase digestion process.^47,48^ The final pH of the mixture was 6.08. Each bottle from the high and low enzyme treatments was spiked with 100 μL of PCB stock mixture (Table S1) and shaken on a horizontal shaker at 150 rpm at 37 °C for 28 days to allow the chemicals to partition to the different components in the gut fluid mixture. This is to mimic an environmentally relevant scenario of chemical intake by humans via both food and MP ingestion. It also mimics an already contaminated human gut due to chemical bioaccumulation in the body over the years. The final concentrations of individual PCB congeners in each system ranged from 30 to 124 μg/L (mass of PCB per volume of assay) (Table S1) to ensure that equilibrium concentrations in plastic and food after 28 days were environmentally realistic.^49^

After 28 days, the POM passive sampler and one LDPE strip were collected from each bottle by using metal forceps. Since partition coefficients for POM passive samplers are accurately known,^39−41^ PCB concentrations in POM are used to determine the PCB concentrations in the aqueous phase. In addition, 200 μL of the gut fluid mixture was sampled for lipid analysis. For the high enzyme treatment, 10 mL of lipase was added to the bottles in its original form based on the enzyme activity concentration from Minekus et al., 2014.^50^ On the other hand, for the low enzyme treatment, 10 mL of lipase was heat-inactivated in a 14 mL glass centrifuge tube by heating it to 100 °C in a water bath for 10 min. The heat-inactivated enzyme was then added to the bottles. This was similarly done for the control setups. The final food (olive oil and LDPE) to gut fluid ratio was 1:10 (v/v)^45,50^ in the systems, with a 1:10 (v/v) ratio for plastic to olive oil. This ratio was chosen to represent digestive systems with a higher volume of lipid in comparison to ingested plastic. The contents of the bottles were mixed by swirling with hands for 5 min before removing one LDPE strip. Subsequently, the gut fluid kinetics experiment was carried out over 72 h with continuous shaking on a horizontal platform shaker incubator at 150 rpm and 37 °C. The experiment was performed for a longer time scale than realistic digestion times. Over longer time scales, higher chemical concentration gradients can be observed; hence, chemical transfer kinetic rates can be assessed more accurately. Furthermore, this way, we also consider possible scenarios whereby MPs are retained in the gut longer than food. Although there is a lack of empirical evidence on the gut retention times for MPs in humans, other studies have shown that the MP retention time in animals’ guts, such as fish and lobsters, differs from food.^51−53^ One LDPE strip was removed after 2, 4, 6, 8, 10, 24, 48, and 72 h of exposure to lipase and then cleaned with a lint-free tissue before storing in acetone-cleaned aluminum foils at −20 °C until chemical extraction. Two hundred microliters of liquid emulsion were collected from the systems after 2, 4, 6, 10, and 72 h of exposure to lipase and immediately transferred into a glass centrifuge tube filled with 2 mL of ternary solvent prepared with dichloromethane, methanol, and MQ-water in a ratio of 1:2:0.8 (v/v/v) for solvent lipid extraction. Chemical analysis of the plastic strips was carried out based on a previously published method.^21^ The methods for lipid extraction and analysis are provided in the SI. The rates of hydrolysis of lipids in the high and low enzyme treatments were calculated using molar concentrations of free fatty acids (FFAs) produced in the test medium over time. The molar concentrations (μM) of FFAs liberated by lipase can be determined directly from the measurements of fatty acids over time or from the change in pH of the systems.^54,55^ The hydrolysis rates indirectly reflect the difference in the enzyme activities between the high and low enzyme treatments.

#### Micelle–Water and Oil–Water Partition Coefficients of PCBs

In addition to the main experimental design, two separate experiments were designed to determine the micelle–water and oil–water partition coefficients of 10 PCBs under varying amounts of micelles and olive oil. Liberated free fatty acids from olive oil can form micelles due to their amphiphilic structure. Therefore, for the micelle experiment, mixed micellar solutions were made with NaTC and increasing additions of oleic acid (i.e., a type of fatty acid) to obtain total micelle-forming surfactant concentrations of 5.7, 6.9, 11.6, and 64.5 g/L in 8 mL of the gut fluid mixture in glass centrifuge tubes containing one POM strip each. The critical micelle concentrations (CMCs) of NaTC and oleic acid are 8–12 mM^56^ and 1.7 mg/L,^57^ respectively. Hence, the concentrations used in this experiment surpassed the CMC, ensuring that micelles were formed. For the olive oil experiment, three levels of olive oil concentrations, 1.3, 6.3, and 12.6 g/L in 8 mL of the gut fluid mixture, were prepared in glass centrifuge tubes also containing one POM strip each. Each system was spiked with 10 μL of the PCB stock mix and shaken at 250 rpm at 37 °C for 28 days, and all experiments were run in quadruplicate. After 28 days, the POM strip was removed from each tube, cleaned with a lint-free tissue, and analyzed for PCBs. The PCB concentrations on the POM strip were then used to calculate the aqueous phase concentrations in the system to estimate the partition coefficients of PCBs for micelles and oil using chemical partitioning theories.^39−41,43,58^ The partition coefficients of PCBs between micelle and water (*K*_micelle_; L/kg) and between oil and water (*K*_oil_; L/kg) can be obtained from the following equations
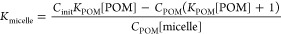
1
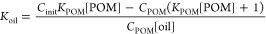
2These equations are obtained by rearranging the chemical mass balance equation of the system (eqs S1–S8 in SI). *C*_init_ is the total initial concentration of each PCB congener in the system (μg/L), *K*_POM_ is the POM-water partition coefficient (L/kg) obtained from Hawthorne et al. (2009),^41^*C*_POM_ is the PCB concentrations in the POM strip normalized to the system volume (μg/L), [POM] is the concentration of POM in the system, i.e., mass of POM per unit of system volume (kg/L), and [micelle] and [oil] are the mass of micelle and oil normalized to the system volume, respectively (kg/L). For a more detailed explanation, the reader is referred to the SI (Determination of partition coefficients *K*_micelle_ and *K*_oil_).

### Model Design, Description, and Implementation

We extended an existing first-order multicompartment model to simulate the exchange of chemicals between plastics, water, micelles, and food.^21^ The model was adapted to include the digestion process of food, and in this case, olive oil was used as a proxy. A schematic overview of the model is provided in SI Figure S1. The PCBs added to our gut fluid mimic system are distributed into the different gut components. Based on chemical mass conservation, the mass of chemicals in μg residing in water (*M*_W_), micelles (*M*_micelle_), oil (*M*_oil_), and the two plastic types in the system, POM (*M*_POM_) and LDPE (*M*_LDPE_), always remains equal to the initial total mass of chemicals (*M*_total_)
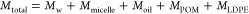
3

If we divide all of the terms in eq 3 by the volume of the system (*V*_w_; L), this yields
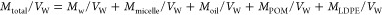
4

Each of these terms thus can be seen as a concentration expressed in terms of the same system volume, which illustrates the conservation of mass in the system
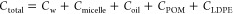
5where *C*_total_ (μg/L) is the initial total concentration of chemicals, *C*_w_ (μg/L) is the chemical concentration in the water, *C*_micelle_ (i.e., aggregates of NaTC and FFA, μg/L), and *C*_oil_ (μg/L) are the concentrations of chemicals in the micelle and oil phase, respectively, and *C*_POM_ (μg/L) and *C*_LDPE_ (μg/L) are concentrations of chemicals in the POM and LDPE strips, respectively, all in terms of the volume of the system (*V*_W_). Note that all of the terms in eq 5 are expressed in μg/L, i.e., normalized to the volume of the system. The terms in eq 5 that relate to micelles, oil, POM, and LDPE can also be expressed in terms of their solid-phase concentrations
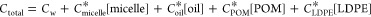
6where *C*_micelle_^*^, *C*_oil_^*^, *C*_POM_^*^, and *C*_LDPE_^*^ are the chemical concentrations in the solid phases (μg/kg), and [micelle], [oil], [POM], and [LDPE] are the concentrations of the solid phases in the system (kg/L). Note that here, the superscript ‘*’ indicates that concentrations are based on solid-phase mass to distinguish them from the concentrations in terms of system volume. At equilibrium, the solid-phase concentrations *C*_micelle_^*^, *C*_oil_^*^, *C*_POM_^*^, and *C*_LDPE_^*^ can be related to the aqueous-phase concentration and the chemical partition coefficients *K*_micelle_, *K*_oil_, *K*_POM_, and *K*_LDPE_ (all with unit L/kg) between each of the phases and water, which leads to
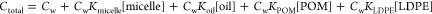
7

Note that all of the terms in eq 7 still have units μg/L and relate to concentrations in different phases in terms of the volume of the system. Equation 7 can be simplified further to
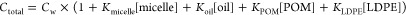
8

It has been demonstrated that 76 μm POM samplers approach equilibrium within 14 days at room temperature.^41^ Hence, the chemical concentration in water at the start of the digestion process (*t* = 0 h), *C*_w_, can be determined from *C*_POM_^*^ after the preincubation of 28 days and *K*_POM_ (L/kg)^39−41,43,59^
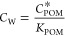
9

Values of *K*_POM_ were derived from a previously established log–linear relationship with *K*_OW_.^41^

Due to the much higher intraparticle diffusivities in both micelles^60^ and oil^61^ as compared to the condensed crystalline POM matrix, and due to the higher temperature, we can safely assume that the chemical concentrations in micelle and oil also reached equilibrium after the preincubation period of 28 days. Therefore, the chemical concentrations in the micelle and oil at the start of the digestion process (*t* = 0 h) can be calculated with *K*_micelle_ and *K*_oil_ established from the experiments defining partitioning coefficients of the micelle and oil, respectively (eqs 1 and 2), with *C*_W_ derived from eq 9
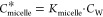
10
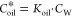
11

**Figure 1:**
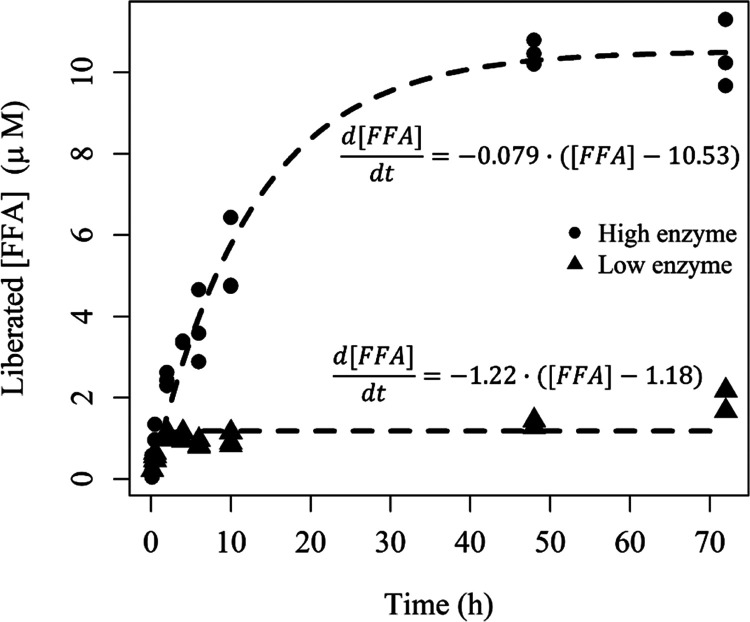
FFA concentration (μM) liberated with time (h) for high and low enzyme treatments based on pH change during lipid digestion.

In the second phase of the experimental setup wherein lipase was added into the systems, a previously described biphasic kinetic model simulating the exchange of chemicals between plastics, water, micelles, and food components^21^ was further developed to include the digestion process. During the hydrolysis of the olive oil triglycerides, FFAs are liberated, which were quantified from the change in pH over time. For each FFA molecule liberated, one proton (H^+^) is released.^54,55^ The FFA molar concentrations display an exponential behavior over time with a limit at infinity (Figure 1). Therefore, the molar concentration of the liberated FFA, [FFA] (μM) over time (h), is fitted with a first-order kinetic rate model^62,63^ (Figure 1)
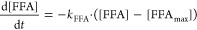
12where *k*_FFA_ is the first-order rate constant (h^–1^) and [FFA_max_] (μM) is the maximum molar concentration of FFA that can be liberated. FFA molecules tend to form micelles due to their amphiphilic structure,^64^ and this thus increases the micellar compartment of the system. The total mixed micelles, [micelle]_*t*_ (kg/L), because of the aggregated FFA and NaTC over time are
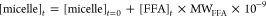
13where MW_FFA_ (g/mol) is the average molecular weight of the FFA based on the fatty acid composition measured (Table S2). On the other hand, while FFA molecules are being liberated, the mass concentration of oil (triglycerides), [oil]_*t*_ (kg/L), decreases over time. This was calculated as
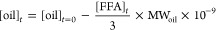
14where MW_oil_ (g/mol) is the molecular weight of olive oil triglycerides, which was calculated from the MW_FFA_ (see eq S13 in the SI). The molar concentration of oil triglycerides is equivalent to one-third of the molar concentration of FFA since a triglyceride (lipid) is made up of three fatty acids.

Following our previous study, for all systems, the boundary condition for *C*_W_ in the second phase of the experiment was calculated by assuming a fast equilibrium redistribution to the mixed micelles, oil, and water with a much slower exchange in the bulk solid polymer (LDPE). Therefore, the LDPE-water chemical exchange is the overall rate-determining process.^21^ The PCB concentration in LDPE (*C*_LDPE_^*^) was modeled by fitting eqs 2 and 3 from Mohamed Nor and Koelmans (2019)^21^ (SI eqs S9 and S10) with the additional aforementioned digestion kinetics, i.e., eqs 8–14. Due to the short time scale of the present experiment (72 h), slow intrapolymer diffusion has little influence, given that the slow compartment kinetic rate constant, *k*_3_ (h^–1^), had a half-life of 13–32 days based on the previous study.^21^ Therefore, only the kinetic rate constants, *k*_1_ and *k*_2_, were fitted using the minpack.lm^65^ package and deSolve^66^ in R. The fast desorption reservoir fraction of the total mass of chemical bound to plastic, *f*_1_ (dimensionless), and the intrapolymer rate constant, *k*_3_ (h^–1^), were set according to obtained relationships with *K*_OW_ from our previous study since the same LDPE strips were used.^21^ Boundary conditions for the PCB concentrations in LDPE were based on the concentrations measured at *t* = 0 h whereby the slow and fast reservoirs of the polymer were assumed to be in equilibrium. The removal of LDPE strips at each time point was also accounted for in the modeling. However, the extraction of the emulsion was not accounted for since the total amount extracted only comprised ∼1% of the total volume of the system.

### Statistical Analysis

To investigate if the different mass concentration levels of micelles and oil influenced the log *K*_micelle_ and log *K*_oil_, respectively, analysis of covariance (ANCOVA) was used to measure the main effect and interaction effects of the mass concentration levels of micelles and oil while controlling the effects of log *K*_OW_. The analysis was performed using ANOVA with Type III sums of squares from the car^67^ package in R.^68^ Prior to the analysis, key assumptions were checked. First, independence between the covariate (log *K*_OW_) and treatment groups (mass concentration levels of micelles and oil) was tested with an ANOVA model. Second, the variances among treatment groups were checked for homogeneity with Levene’s test found in the car^67^ package. Since the mass concentration levels of micelles and oil had a significant effect while controlling the log *K*_OW_ variable, a post hoc multiple comparisons was performed using the Tukey test (multcomp package^69^) to determine which treatment groups are different from each other. ANCOVA was similarly performed to investigate the influence of the enzyme treatments on log *k*_1_ and log *k*_2_ while controlling the effects of log *K*_OW_. ANCOVA was also used to investigate the differences between the linear regressions (log *K*_micelle_ vs log *K*_OW_) of Schacht et al. (2016)^70^ and our study.

## Results and Discussion

### Sorption Affinities of Olive Oil Triglycerides and Micelles

**Figure 2:**
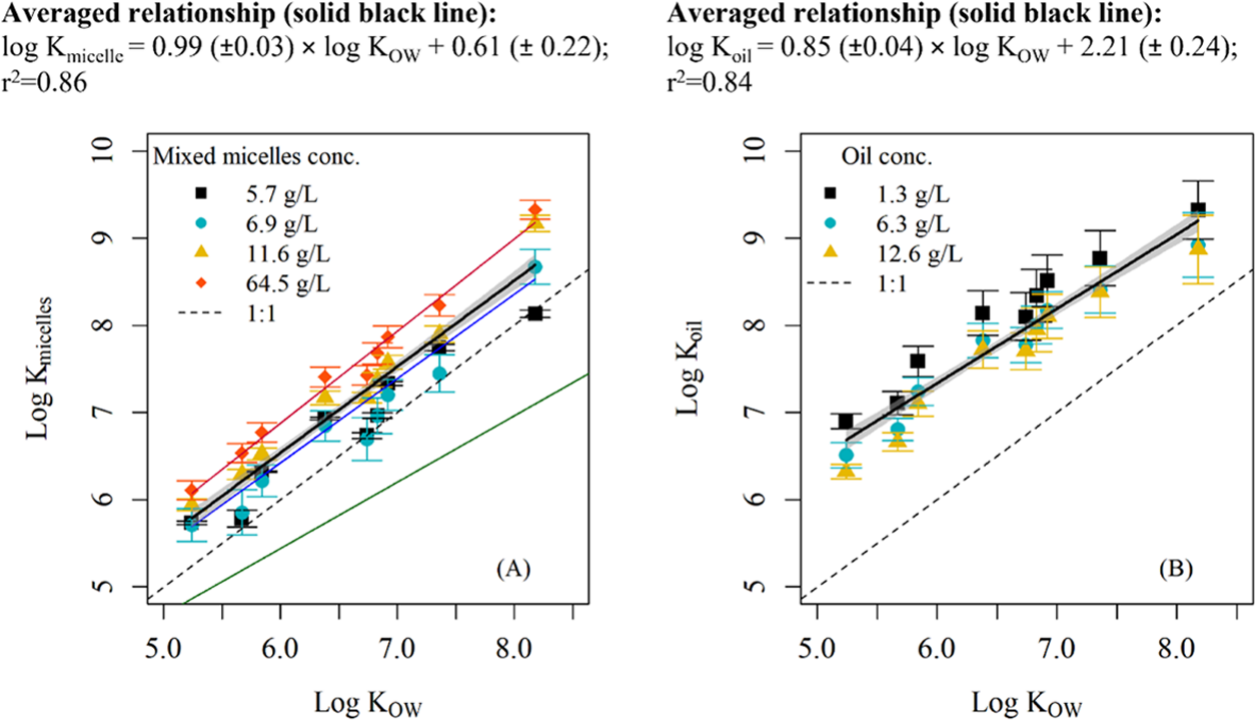
(A) Log micelle–water distribution coefficients (*K*_micelles_) vs log *K*_OW_ for 4 concentration levels of micelle-forming surfactant concentrations (NaTC and FFA). The red line is the linear regression fitted for the highest concentration (64.5 g/L), the blue line is the linear regression fitted for the other concentration levels (5.7, 6.9, 11.6 g/L), and the black line is the linear regression fitted for all concentrations with a 95% confidence interval indicated by the gray band. Regression equations are reported in Table S5. The solid green line is the linear relationship obtained by Schacht et al., 2016. (B) Log oil–water distribution coefficients (*K*_oil_) vs log *K*_OW_ for 3 oil concentrations (1.3, 6.3, 12.6 g/L) (Table S7). The solid black line is the linear regression fitted for all concentrations with a 95% confidence interval.

Due to the dynamic nature of the human digestive system, the concentrations of lipids and micelles change over time as lipids break down. Since the FFA molecules produced during the hydrolysis process would form micelles in the gut fluid,^71,72^ the effect of increasing concentration of micelle-forming surfactants (NaTC and FFA) on the micelle–water distribution coefficients of the combined mixture of micelles was investigated. The lowest micelle concentration (5.7 g/L) was the baseline of the gut fluid system, whereby only NaTC formed micelles. Oleic acid was added to the other three treatments to achieve the respective micelle-forming surfactant concentrations. This mimics the dynamic behavior of the main experiment in which more fatty acids were produced over time as lipid digestion occurred. The different mass concentrations of mixed micelles in this study had a significant influence on the micelle–water partition coefficient (Figure 2A) after controlling for the effect of log *K*_OW_, *F*(3,139) = 81.86, *p* < 0.001 (SI Table S4). The post hoc pairwise comparisons revealed that all treatments differed from each other except the two lowest mass concentration treatment groups (5.7 and 6.9 g/L). However, the differences of the micelle–water partition coefficients were generally less than 1 order of magnitude for the less hydrophobic PCBs. Since the variability of the log *K*_micelles_ was less than 1 log unit, an average linear relationship between log *K*_micelle_ and log *K*_OW_ with a slope of 1 across all mixed micelle concentrations was determined (log *K*_micelle_ = 0.99 (±0.03) × log K_OW_ + 0.61 (±0.22); *r*^2^ = 0.86). Our linear regression was significantly different from the relationship established by Schacht et al., 2016^70^ (ANCOVA; *p* < 0.001; Table S3), as shown by the solid green line in Figure 2A. This implies that the mixture of micelle-forming surfactants from NaTC and FFA and even the NaTC alone behave differently from sodium dodecyl sulfate (NaDS), which was used in the aforementioned study. Due to its aggregation properties and physical characteristics, NaTC forms smaller aggregates and can solubilize chemical molecules from the aqueous phase more easily than NaDS.^73^

The effect of oil concentrations on the oil–water partition coefficients across the range of PCBs in the present study was also significant, *F*(2,104) = 29.60, *p* < 0.001 (SI Table S6 and Figure 2B). The lowest oil concentration level (1.3 g/L) was significantly different from the other two treatment groups (*p* < 0.001; Tukey test). However, the regression coefficients of each treatment group also did not differ by more than 1 log unit. The limited differences in the sorption partition coefficients for PCBs in the olive oil phase at different concentrations (1.3–12.6 g/L) suggest that there was no significant change in the sorption behavior at different oil concentrations. Therefore, an average log–linear relationship was defined for all three olive oil concentration levels (log *K*_oil_ = 0.85 (±0.04) × log *K*_OW_ + 2.21 (±0.24); *r*^2^ = 0.84). In general, *K*_oil_ values were significantly higher than *K*_OW_ values by an average factor of 16 (Figure 2B). The results shown corroborated an earlier study on the sorption of PCBs to gas oil and crude oil, which found *K*_oil_ values 7 times higher than *K*_OW_ values.^61^

The mixed micelles have lower partition coefficients than olive oil. Yet, they are higher than the LDPE-water distribution coefficients, which are determined from the transfer rate kinetics in this study (see the following section and Figure S5). Therefore, the micelle compartment has a higher sorption partition coefficient and affinity than LDPE but lower than olive oil (*K*_oil_ > *K*_micelles_ > *K*_LDPE_).

### MP Sorbs PCBs Released from Food Digestion

Lipid digestion was demonstrated by the increase in the number of hydrogen protons liberated over time with the release of FFA (Figure 1). Measurement of FFA released based on pH^54^ is an adequate representation of the lipid digestion process as this method is direct and not influenced by the possibility of continued hydrolysis after the emulsion is extracted. Based on Tan et al., 2020,^32^ lipid digestion occurs rapidly during the first 2 h of the digestion process. Therefore, the analysis of lipid digestion via lipid extraction is less accurate when the enzyme reaction kinetics are measured over time. Nevertheless, the triglyceride concentration measured via lipid extraction and gas-chromatography analysis showed a general decreasing trend over time (Figure S3) though the trends were indistinguishable for the high enzyme treatment and the denatured enzyme treatments (i.e., low enzyme and control). This supports our earlier suggestion that hydrolysis may have continued to occur after the emulsion samples were extracted since lipase was still present in the emulsion.

The release of FFA in the high enzyme treatment differed significantly from that in the low enzyme treatment (Figure 1). Both demonstrated an exponential behavior approaching a limit within the 72 h experimental time period. The liberated protons over time were fitted with eq 12, and the first-order rate constants, *k*_FFA_, and maximum FFA limit, [FFA_max_], are shown in Table 1.

**Table 1:** Parameter Estimates for High and Low Enzymatic Activities Were Based on pH Measurements over Time. Standard errors are in parentheses

	[FFA_max_] (μM)	*k*_FFA_ (h^–1^)
high enzyme	10.53 (0.32)***	0.079 (0.007)***
low enzyme	1.18 (0.09)***	1.22 (0.52)*

***Significant at *p* < 0.001; **significant at *p* < 0.01; *significant at *p* < 0.05.

In the first 2 h of the digestion process, the initial rates of FFA released were not distinguishable between the high and low enzyme treatments. However, both enzyme treatments showed a rapid increase in FFA initially, which agrees with an earlier study.^32^ The first-order rate constant (*k*_FFA_) in the low enzyme treatment is about 15 times higher than the *k*_FFA_ for the high enzyme treatment, despite the lower enzyme concentration due to the denaturing process. This is contrary to the generally confirmed theory that enzyme rate kinetics would increase with an increasing enzyme concentration. We argue that the fitted rate constant for the low enzymatic activity does not well represent the actual enzyme kinetics due to the lack of data within the first 2 h of the reaction. This is also further supported by the less statistically significant *k*_FFA_ in the low enzyme activity treatment (*p* = 0.029) as compared to the rate constant in the high enzyme activity. The [FFA_max_] was 9 times higher for the high enzyme treatment than that for the low enzyme treatment. This significant difference implies that 9 times more FFAs were liberated for the high enzyme treatment. It is also important to note that the digestion process may be affected by the concentration of MPs in the gut fluid. A recent study observed that lipid digestion decreased with an increasing polystyrene MP concentration as the particles heteroaggregated with lipid droplets and also acted as a sorbent for lipase, altering its secondary structure.^32^ Therefore, the enzyme digestion parameters fitted here apply only to the digestive conditions of the present study.

**Figure 3:**
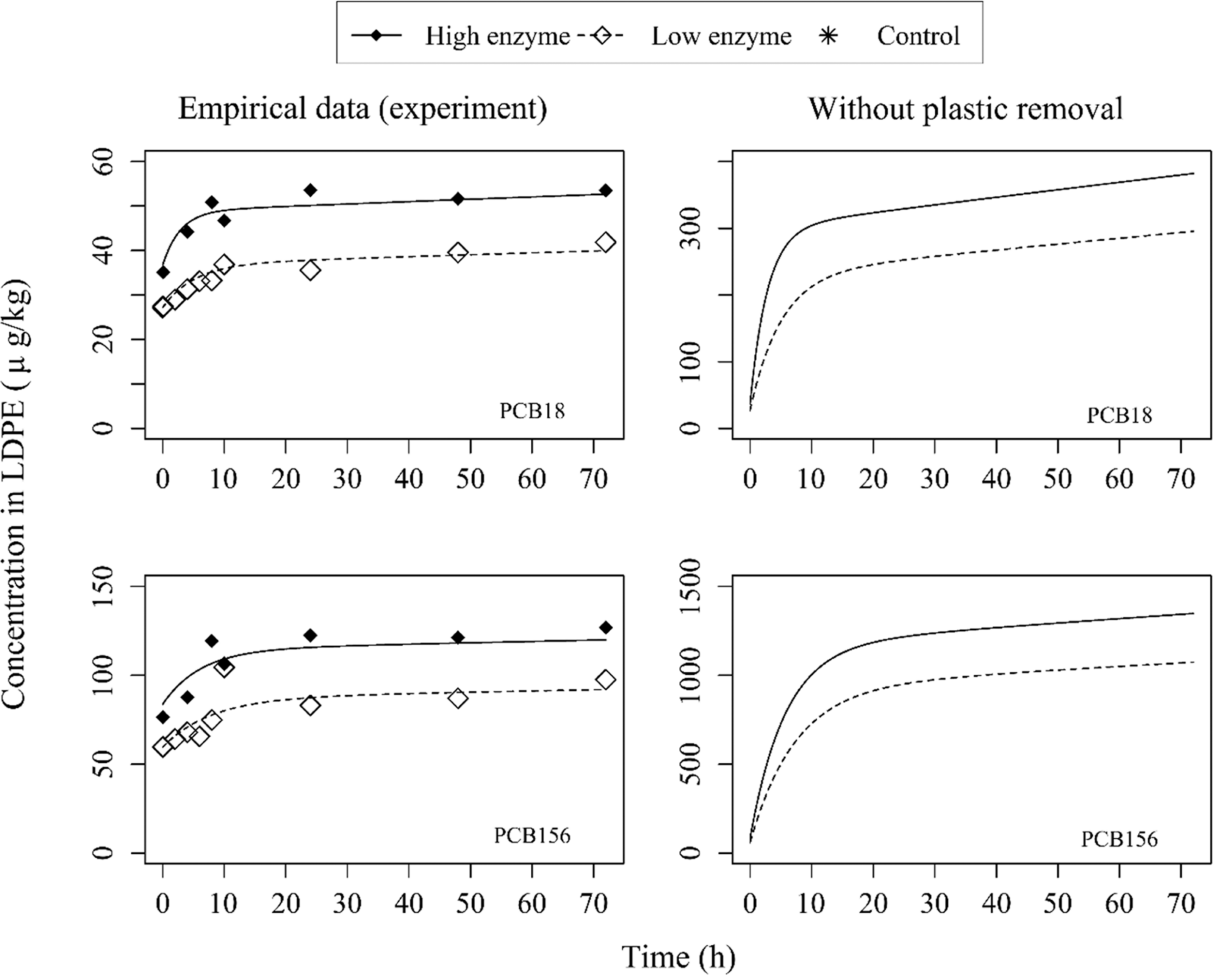
PCB concentrations (PCB18 and 156) in the plastic compartment (μg/kg) over time (h) for high and low enzyme treatments and control (no PCBs spiked) of one replicate system (for all replicate systems, refer to the SI). PCB concentrations were below detection limits (see the SI) in the control systems and are therefore not reflected in the figures. Solid lines represent the fitted models (eqs 3 and 14) for the high enzyme treatment, whereas the dashed lines represent the fitted models for the low enzyme treatment. Left panels show empirical data based on experiment conditions. Right panels show simulated data for the environmentally realistic exposure scenario without the removal of plastic strips.

The hydrolysis of olive oil triglycerides leads to two major implications for the digestive system. First, during the hydrolysis process, sorbed PCBs in the “food” olive oil are released into the aqueous phase to repartition in the other gut compartments and move toward a new equilibrium as the system changes over time.^74^ Second, the FFA molecules produced from the hydrolysis process would form micelles in the gut fluid and thus increase the total micelle concentration for solubilization of PCBs.^71,72,74^ We observed a substantial increase in the PCB concentrations in LDPE over time during the lipid digestion process (Figures 3 and S4). This indicates that PCBs sorbed in our food proxy, i.e., the olive oil triglyceride fraction, were released during lipid digestion and repartitioned in the gut fluid system, partially sorbing onto the LDPE strips. In other words, this demonstrates that MP particles can sorb chemicals from contaminated food during digestion. Here, the percentage increase in the PCBs bounded to the LDPE was not significantly different between the high and low enzyme treatments within 72 h (Figure S6). This is because LDPE represented less than 10% of the main gut fluid components (i.e., micelles, oil, LDPE, and POM) in terms of mass concentration, which even reduces over time due to the removal of plastic strips for PCB measurement, whereas olive oil made up 80%. Furthermore, as discussed earlier, the LDPE in this study has a sorption partition coefficient lower than that of micelles and olive oil. As the oil breaks down and releases the sorbed contaminants, the micelles compete with LDPE for the uptake of PCBs. Hence, we observed only a small difference of about 30–80% in the concentration of PCBs (i.e., μg/kg plastic) taken up by the polymer between the high and low enzyme treatments in the experimental setup (Figure S6). However, in nature, microplastic mainly remains in the gut during digestion, as it cannot be easily absorbed. Therefore, we used the model parameters based on the experiment conditions to simulate the environmentally realistic exposure scenario that plastic strips were not removed from the systems (Figure 3, right panel). Since the amount of plastic and total concentration of PCBs in the system remain constant over time, we observe a much larger increase in the concentration of PCBs in the LDPE by a factor of about 10–20.

### PCBs Transfer Kinetics during Hydrolysis of Triglycerides

We investigated an environmentally relevant scenario of the human digestive tract; a human has bioaccumulated contaminants and ingests contaminated food and contaminated plastic.^9^ This was mimicked by pre-equilibrating the contaminants in the gut fluid before the system is exposed to enzymes that can alter the composition of the gut fluid. Lipase is then added to break down the olive oil triglycerides, which would release the lipid-bound PCBs into the gut fluids. We hypothesized that these released PCBs would repartition into the other gut compartments, including LDPE, thereby increasing the PCBs associated with LDPE. The chemical transfer rate kinetics in the LDPE may be affected by the enzyme digestion process, depending on which process is rate-limiting (plastic sorption versus enzyme kinetics).

As mentioned, the experiment was performed for a longer period than the standard upper gastrointestinal tract retention time. The PCB concentrations increased rapidly for the first 10 h and then approached a pseudoequilibrium state within 72 h. Previously, we observed biphasic chemical transfer kinetics during a 28 day exposure period.^21^ However, in the present study, this biphasic behavior was not apparent due to the shorter time scale (Figure 3). Therefore, only the fast sorption and desorption kinetic rate constants of the biphasic reversible model (see the SI for more details) were fitted to the high and low enzyme treatments (Table S8). For the control treatment, only PCB 52 and PCB 101 showed a slight increase in concentration in LDPE over time. The levels of contamination were close to the minimum detection limit and similar to the extraction method blank (Table S1 in the SI). Therefore, this contamination was not accounted for in the respective high and low enzyme treatments as the level of contamination was low, and the data still fitted well with the model and initial boundary conditions set by the measured PCB concentrations in the POM strip. PCB77 and 169 were omitted from further analysis due to high relative standard deviations in the response factors of replicate measurements of calibration standards.

**Figure 4:**
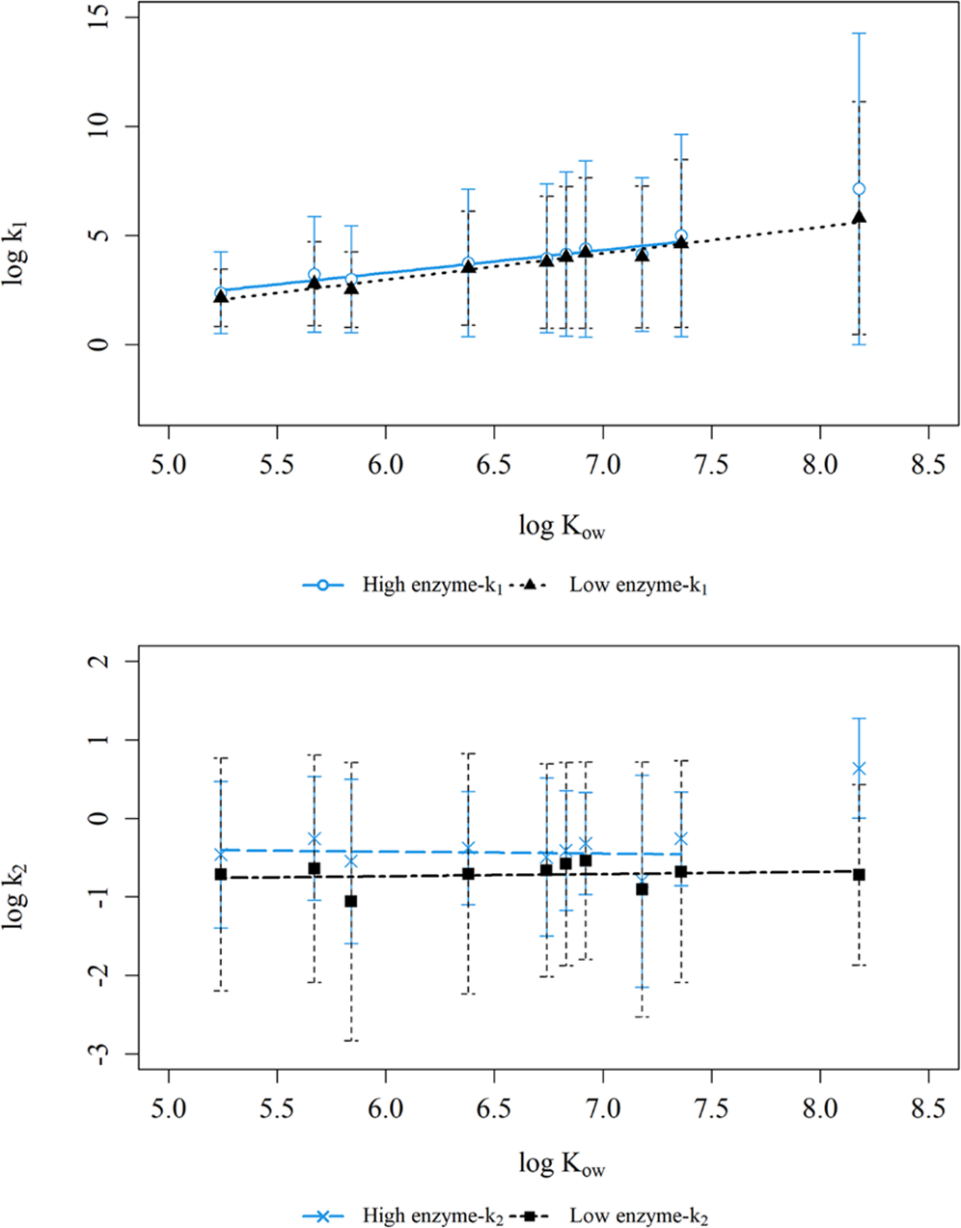
Log values of kinetic parameters (top panel: *k*_1_; bottom panel:*k*_2_) vs log *K*_OW_ for low and high enzyme treatments. The error bars represent the standard errors from 4 replicate systems.

The fast sorption rate constants (*k*_1_) were statistically significant for all PCBs in both enzyme treatments, except PCB 209 (Table S8). They ranged from 236 to 1.37 × 10^7^ h^–1^ and 138 to 3.77 × 10^4^ h^–1^ for high and low enzyme treatments, respectively. The fitted *k*_1_ values for some congeners here were about 2−8 times higher than those from a similar chemical exchange kinetics experiment involving MP and food but without food digestion kinetics, in which LDPE strips from the same source were used.^21^ The log *k*_1_ values were significantly positively correlated with log KOW in both high (log *k*_1_ = 1.04 (±0.11) × log *K*_OW_ − 3.01 (±0.73), *r*^2^ = 0.87, *n* = 10) and low (log *k*_1_ = 1.21 (±0.08) × log *K*_OW_ − 4.28 (±0.51), *r*^2^ = 0.96, *n* = 10) enzyme treatments (Figure 4). The positive correlations corroborated with the earlier study in the presence of organic matter.^21^ The slope coefficients in the regressions for *k*_1_ in this study are higher than the earlier study^21^ with organic matter. This means that the uptake transfer rates of PCBs from the water phase to LDPE increase more as the hydrophobicity of the compound increases. We speculate that the facilitated transport by the oil in this study has a more significant influence on the uptake of PCBs than on organic matter. This is further supported by the oil−water partition coefficients that are 2 orders of magnitude higher than organic matter-water partition coefficients.^21,79^ Although the theory of facilitated adsorption for oil and organic matter is well-established in the field of environmental pollution (e.g., (78,80−82)), little is known about the effects of vegetable oil or other food matrices in the cotransport of contaminants across the aqueous boundary layer to indigestible materials such as plastic particles in the gut fluid. Besides the co-transport of contaminants through oil, the NaTC may also play a role in enhancing the uptake of PCBs onto the LDPE. The presence of NaTC in the gut fluid also acts as an oil dispersant, which facilitates the formation of oil-surfactant aggregates. These dispersed aggregates can promote the uptake of oil-associated contaminants onto the LDPE.^78^ The enzyme treatments in this study had a significant influence on the uptake kinetic rate constant, *k*_1_, after controlling for the effect of log *K*_OW_, *F*(1,16) = 5.19, *p* = 0.04 (SI Table S10). This may be due to the higher amount of FFAs released in the high enzyme treatment than the low enzyme treatment, which may also facilitate the uptake of PCBs onto the LDPE.^59,75−78^

The reverse transfer kinetic (desorption) was also included in our model since chemical transfer occurs in both directions simultaneously as demonstrated earlier.^21^ The desorption rate constants (*k*_2_) of the fast fraction were statistically significant for all PCBs in both treatments except PCB 209 in the high enzyme treatment (Table S8). In the high enzyme treatment, *k*_2_ ranged from 0.283 to 4.36 h^–1^, translating to desorption half-lives of 0.16–2.45 h. On the other hand, fitted *k*_2_ values in the low enzyme treatment were significantly lower than the high enzyme treatment, *F*(1,16) = 13.82, *p* < 0.01 (SI Table S10), ranging from 0.087 to 0.289 h^–1^, translating to desorption half-lives of 2.40–7.97 h. Furthermore, they were in a similar range to the estimated *k*_2_ values from our aforementioned study, which investigated the chemical kinetics of plastic with an inert food component.^21^ Unlike the uptake rate constants (*k*_1_), the desorption rate constants (*k*_2_) for the high and low treatments had weak positive correlations with hydrophobicity (Table S9), suggesting that the desorption process was not influenced by the bulk size or hydrophobicity of the PCB congeners. Both the uptake and desorption rate constants were influenced by the enzyme digestion process. The high enzyme treatment results in a higher formation rate of FFA molecules, which may be driving the PCB transfer in/out of the LDPE via facilitated transport. Here, facilitated transport refers to the additional transport of chemicals between the aqueous phase and the LDPE due to the presence of organic molecules in the diffusive boundary layer around the LDPE.^29,30^

LDPE distribution coefficients (*K*_P_) were derived from the fitted *k*_1_ and *k*_2_ estimates (eq S14 and Figure S5). The log *K*_P_ positively correlated with log *K*_OW_ (log *K*_P_ = 1.04 (±0.03) × log *K*_OW_ + 0.05 (±0.23); *r*^2^ = 0.98, *n* = 20) with a slope close to 1, corroborating previous studies.^83−86^

### Percentage Reduction in Chemical Bioavailability

The chemical concentrations bound to LDPE after the pre-equilibration period and before the lipid digestion process, comprised 0.2–4% of the total concentrations in the system. These percentages depended on the hydrophobicity of the compound, with more hydrophobic compounds having higher percentages of PCB bound to LDPE. This was largely due to the higher slope coefficient of the log *K*_P_ – log *K*_OW_ regression for LDPE, implying a larger increase in *K*_P_ as log *K*_OW_ increases.

After the lipid digestion was initiated for 72 h, LDPE took up part of the PCBs released from the digested olive oil triglycerides, resulting in an up to 75% increase in PCB concentrations in the LDPE (Figures 3, S4, and S6). This implies that MP particles leaving the body would have substantially higher chemical concentrations than when entering the body, which may seem counterintuitive yet logical based on the processes at play. This increase, however, still implies a decrease in chemical bioavailability by about 0.11–0.87% (Figure S7) of the total chemical amount present in the gut. This low percentage is mainly due to the removal of plastic strips during the experiment, which significantly reduces the amount of PCBs taken up by the plastic phase. As mentioned earlier, in reality, ingested microplastics are typically not absorbed by the gut and thus remain in the gut during digestion.^29^ We used the kinetic parameters based on the empirical data to simulate the percentage reduction in bioavailability for the environmentally realistic scenario that the plastic remains in the gut over time (Figure S8). The results show that LDPE can reduce bioavailability by 3–40% as lipids are digested. Furthermore, the percentage distribution of PCBs in the LDPE phase increases by about 3–10% after 72 h of lipase digestion (Figure S10). This implies that fewer PCBs are absorbed by the body via the FFAs and micelles as LDPE takes up a higher fraction of the PCBs released by the lipids during digestion.

We also observed a noticeable increase in the percentage reduction in the chemical bioavailability for more hydrophobic compounds (Figures S7 and S8). The overall reduction in chemical bioavailability is limited by the digestion rate kinetics, which is slower than the uptake kinetics (*k*_1_). Furthermore, LDPE is competing with micelles, which have about 1.5–2 times stronger binding affinity than LDPE across the log *K*_OW_ range of PCBs tested in this study.

The observed percentage reduction in chemical bioavailability demonstrated in the present study is low, as the chemical uptake is also driven by the chemical fugacity of the LDPE at the start of the digestion process. Our earlier study simulating gut fluid conditions with clean plastic (i.e., hence, lower fugacity than in the current study) and contaminated organic matter as food demonstrated that it is possible for MPs to clean the gut up to almost 90% without digestion if the MPs remain in the gut for more than 28 days and are eventually excreted out in the feces.^21^ In addition, the mass ratio of LDPE to food used in this study is 1:10. This ratio is lower than what was implemented in the earlier study^21^ to ensure that the mass ratio of LDPE to food is more relevant to what is found in nature.^87,88^

Additionally, despite the difference in the exchange kinetics, the overall change in chemical bioavailability did not differ significantly between the high and low enzyme treatments. This implies that the digestion dynamics will not result in significant differences in the chemical bioavailability when the plastic-to-food ratio in the gut is similar to that in the present study and also equally contaminated (at near-equilibrium). Therefore, here we show that a contaminated LDPE is still capable of further taking up part of the released PCBs from food during digestion but with little effect on the digestion dynamics. Furthermore, the speculated facilitated transport attributed to the surrounding oil and free fatty acids enhances the partitioning of PCBs on LDPE, potentially making it a relevant chemical “cleaning” agent for ingested contaminated food. This similar “cleaning” phenomenon had been demonstrated earlier with olestra, a nondigestible lipophilic dietary fat (similar to the LDPE here), which could take up 2, 3, 7, 8-tetrachlorodibenzo-*p*-dioxin (TCDD) and reduce TCDD concentrations.^89−92^

### General Discussion of Limitations and Implications

We presented an *in vitro* experimental design and a chemical kinetic model to investigate the chemical dynamics of MPs under simulated gut conditions, including intestinal digestion processes. The model enables the accurate parameterization of kinetic parameters to describe the chemical transfer with MPs and digestion dynamics, which is crucial given the growing concerns regarding the chemical impacts of MPs. Our experimental setup replicated a scenario where aquatic organisms or the human gastrointestinal tracts (GITs) are exposed to contaminated food and plastic through ingestion. We acknowledge that other scenarios, such as contaminated food and clean plastic or clean food and contaminated plastic, have been previously discussed in the literature.^21,29,30,93^ Importantly, our chemical kinetic parameters can also be applied to model the chemical behavior of HOCs with LDPE in other gut scenarios, such as different chemical concentrations, gut transit times, or variations in MP, lipid, micelle, and food mass concentrations. These results can be extrapolated to assess the exposure and risks of plastic-associated HOCs.^9,93^

We acknowledge that our experimental study did not account for other dynamic changes such as the absorption of micelles and their contents, such as short-chain fatty acids, across the gut lumen, as this was not a primary focus of our study. However, the chemical kinetic model framework and conceptual findings from our study are also applicable to other dynamic gut systems for aquatic organisms and, thus, aquatic food webs. This is because sodium taurocholate, a vertebrate bile salt used in our study to represent micelles in the gut, has been widely used to mimic intestinal fluids of aquatic species (e.g.,^20,94−96^). We used lipids from olive oil as a proxy for food and LDPE as a proxy for MPs at specific concentrations. Further studies are needed to investigate other types of food and different ratios of MP to food concentrations since it has been demonstrated that MPs can also hinder digestion.^32^

Our study focused on the environmentally relevant scenario where chemicals in food and MPs have comparable fugacities. The change in food composition, specifically the hydrolysis of triglycerides to free fatty acids (FFAs) during digestion, increased the chemical fugacity in the gut. This increase serves as the driving force for biomagnification in nature. In aquatic exposures, for instance, in the absence of microplastic ingestion, it is realistic to consider biomagnification reaching a steady state for fish or invertebrate species native to the contaminated aquatic environment. These organisms are already in chemical equilibrium (or at steady state) from the egg or larval stage even before they start ingesting microplastic particles. In this scenario, when MPs are ingested, the increased fugacity in the digestive tract available for biomagnification leads to uptake by the ingested microplastic particles as well (see Figure 3), thus attenuating biomagnification.^21,31^ MPs, due to their strong affinity for HOCs, absorb a fraction of these chemicals and act as a sink. While chemicals absorbed by micelles would pass through the gut lumen into the bloodstream in a real gut, the chemicals bound to MPs are not absorbed by the body, unless they can pass through the gut lining. As a result of the subsequent egestion of contaminated MPs, there is reduced availability for the transfer of chemicals across the gut lining compared to the scenario without microplastic ingestion. Therefore, our results empirically demonstrate that MPs can decrease chemical bioavailability during food digestion, a phenomenon consistent with the prevailing biomagnification theory.^26,27^ It is important to note that the reduction in the bioavailability of PCBs due to the nonabsorbable MPs is limited by the digestion kinetics of lipids, which has a half-life that is 200 times longer than the uptake kinetics of PCBs by LDPE.

The above discussion pertains to the fraction of MP particles that are too large to pass through the gut lining and be absorbed by the body. It has been suggested that a fraction of contaminated MPs may be small enough to pass through the gut lumen.^9,97^ In such cases, the increase in sorbed chemical concentrations in MPs could result in a particle-mediated transfer. However, this is only relevant to the very smallest particles (e.g., <10 μm), which represent a marginal weight fraction of the entire ingested MP size continuum.^98^ Furthermore, these particles are assumed to have a very low absorption efficiency of approximately 0.3% based on empirical data.^9^ Calculation shows that the mass of chemicals transported via this pathway is minimal compared to the mass of chemicals absorbed from the gut by larger (>10 μm) particles due to the digestion of food, which are subsequently egested (calculation provided in the SI). Thus, the net effect is that MPs remove HOCs from the biota under the environmentally relevant scenario studied.

As mentioned earlier, the conclusions of our study are applicable only to scenarios whereby MPs are more or less equally contaminated as food. It is important to highlight that if MPs are more highly contaminated than food (e.g., plastic additives), thermodynamic principles suggest that the chemicals would transfer to the lower fugacity compartment of the gut. Consequently, in such a scenario, MPs would not reduce the chemical bioavailability during the digestion process.

## Supporting Information Available

Details of PCB congeners and concentrations used in the experiment; description of lipid extraction and separation method; schematic diagram of multicompartment model including digestion dynamics; picture of the gut fluid setup; description on the determination of distribution coefficients for micelle and oil; biphasic sorption on the microplastic model and other auxiliary equations; concentration of triglycerides over time; average percentages of major fatty acids found in olive oil; analysis of covariance for micelle and oil; post hoc multiple pairwise comparison for K_micelle_; linear regression between log *K*_micelle_ and log *K*_OW_; post hoc multiple pairwise comparison for *K*_oil_; linear regression between log *K*_oil_ and log *K*_OW_; total PCB concentrations in LDPE over time after lipid digestion initiation; kinetic rate constants of LDPE for high and low enzyme treatments; linear regression models of **k**_1_ and **k**_2_ for high and low enzyme treatments; analysis of covariance for **k**_1_ and **k**_2_; relationship between log *K*_P_ and log *K*_OW_; percentage increase in LDPE after 72 h of lipase digestion; percentage reduction in the bioavailability of each PCB congener (empirical); percentage reduction in the bioavailability of each PCB congener (env. realistic); percentage distribution of PCB18 and PCB156 in each compartment (empirical); and the percentage distribution of PCB18 and PCB156 (env. realistic) (PDF)
