# Supplementary material for: How Digestive Processes Can Affect the Bioavailability of PCBs Associated with Microplastics: A Modeling Study Supported by Empirical Data
Source: Environ Sci Technol. 2023 Jul 28;57(31):11452–64. doi: 10.1021/acs.est.3c02129 (PMC10413949; doi:10.1021/acs.est.3c02129)
Supplement: Supplementary file 1 — es3c02129_si_001.pdf [file es3c02129_si_001.pdf]

Supporting Information for

How digestive processes can affect bioavailability of PCBs associated with microplastics. A modelling study supported by empirical data.

Nur Hazimah Mohamed Nor, Zhiyue Niu, Marie Hennebelle, Albert A. Koelmans

This Supporting Information contains 24 pages, 10 Figures, 10 Tables

Table S1– Details of PCB congeners and concentrations in spike stock mixture and experiment set-up

Lipid extraction and separation method

Figure S1– Schematic diagram of the multi-compartment model including digestion dynamics.

Figure S2 – Picture of gut fluid setup

Determination of distribution coefficients $K_{micelle}$ and K_{oil}

Biphasic sorption on microplastic model

Auxiliary equations

Calculation of chemical contribution from small MP particles (<10 μm)

Figure S3– Concentration of triglycerides over time

Table S2– Average percentages of major fatty acids found in olive oil

Table S3– Analysis of covariance (ANCOVA) to analyze the influence of different levels of micelle and oil mass concentrations on the partition coefficients, $K_{micelle}$ and K_{oil}, respectively over the log K_{OW} (covariate) range using the Type III test

Table S4– Post-hoc multiple pairwise comparisons using Tukey technique for $K_{micelle}$.

Table S5– Linear regressions between log $K_{micelle}$ and log K_{OW}

Table S6– Post-hoc multiple pairwise comparisons using Tukey technique for K_{oil}.

Table S7– Linear regressions between log K_{oil} and log K_{OW}

Figure S4– Total PCB concentrations in LDPE over time after initiation of lipid digestion

Table S8– Kinetic rate constants of LDPE for high and low enzyme treatments

Table S9– Linear regression models of k_1 and k_2 for high and low enzyme treatments

Table S10– Analysis of covariance (ANCOVA) to analyze the influence of enzyme treatments on the transfer kinetic rate constants, k_1 and k_2, respectively over the log K_{OW} (covariate) range using the Type III test.

Figure S5– Relationship between log K_P and log K_{OW}

Figure S6– Percentage increase in LDPE after 72h of lipase digestion

Figure S7– Percentage reduction in bioavailability of each PCB congener (empirical)

Figure S8– Percentage reduction in bioavailability of each PCB congener (env. realistic)

Figure S9– Percentage distribution of the PCB18 and PCB156 in each compartment (empirical)
Figure S10– Percentage distribution of the PCB18 and PCB156 in each compartment (env. realistic)

Table S1. Details of PCB congeners and concentrations in spike stock mixture and experiment set-up

Congener	Chlorine	Molecular formula	Molecular weight (g/mol)	Planarity	Log K_{OW}[1]	Spike mixture concentration (mg/L)	Concentration in main experimental set-up (µg/L)	Minimum Detection Limit (MDL)[2] (µg)	LDPE Method Blank (µg)
PCB 18	Tri	$C_{12}H_7Cl_3$	257.5	Non-planar	5.24	23.2	29.57	0.0004	<MDL
PCB 28	Tri	$C_{12}H_7Cl_3$	257.5	Non-planar	5.67	42.0	53.57	0.0017	<MDL
PCB 52	Tetra	$C_{12}H_6Cl_4$	292	Non-planar	5.84	73.1	93.25	0.0017	<MDL
PCB 77[a]	Tetra	$C_{12}H_6Cl_4$	292	Planar	6.36	23.9	30.46	0.0044	<MDL
PCB 101	Penta	$C_{12}H_5Cl_5$	326.4	Non-planar	6.38	97.2	123.99	0.0008	<MDL
PCB 118	Penta	$C_{12}H_5Cl_5$	326.4	Non-planar	6.74	36.0	45.95	0.0006	<MDL
PCB 138	Hexa	$C_{12}H_4Cl_6$	360.9	Non-planar	6.83	54.0	68.90	0.0007	<MDL
PCB 153	Hexa	$C_{12}H_4Cl_6$	360.9	Non-planar	6.92	74.6	95.17	0.0007	<MDL
PCB 156	Hexa	$C_{12}H_4Cl_6$	360.9	Non-planar	7.18	34.5	43.96	0.0005	<MDL
PCB 169[a]	Hexa	$C_{12}H_4Cl_6$	360.9	Planar	7.42	39.2	50.06	0.0003	<MDL
PCB 180	Hepta	$C_{12}H_3Cl_7$	395.3	Non-planar	7.36	81.1	103.49	0.0007	<MDL
PCB 209	Deca	$C_{12}Cl_{10}$	498.6	Non-planar	8.18	58.3	74.43	0.0004	<MDL

[1] Hawker and Connell, 1988
[2] Minimum detection limit is based on the lowest detectable concentration on the calibration curve
[a] PCB 77 and 169 had high relative standard deviations (> 15%) for the response factors of replicate measurements of the calibration standards. Therefore, they were omitted from further analysis.

Lipid extraction and separation

The method for lipid extraction was adapted from Paik et al., 2009. Two hundred μL of sample lipid emulsion was vortexed with 2 mL ternary solvent (DCM:MeOH:H_2O=1:2:0.8; v/v/v) briefly. Additional dichloromethane (DCM) and MQ-water were added to the extracts to adjust the ratio of DCM:MeOH:H_2O to 1:1:0.9 (v/v/v). The extracts were then separated by centrifugation at 2500 ×g for 15 mins. The upper layer was pipetted out and the remaining DCM layer was dried over anhydrous Na_2SO_4 and then evaporated to dryness with a gentle stream of nitrogen. The extracts were then diluted in 12 mL hexane and then stored in -80°C until further extraction was carried out.

Lipid extracts were thawed to room temperature and concentrated to 1 mL with a speed vacuum for 15 mins. The triglycerides and free fatty acids were separated based on an adapted method from Richardson et al., 2017. Briefly, 1 mL of cold distilled water was added to the concentrated lipid extracts (1 mL) and vortexed. Then 1 mL of 0.4 M NaOH in MeOH was added and vortexed for 10s. This would allow the NaOH to react with the free fatty acids to form salts. Three mL of hexane were added immediately after vortexing to separate esterified fatty acids then vortexed again. The emulsion was then left to separate for 3 min before the hexane upper phase (contains esterified triglycerides) is transferred into a clean centrifuge glass tube and dried under a gentle stream of nitrogen to 20 μL. The esterified triglycerides were then derivatized to obtain fatty acid methylated esters (FAMEs) (Richardson et al., 2017). Briefly, 20 μL of esterified triglycerides extract was mixed with 400 μL toluene, 3 mL MeOH and 600 μL of 8% HCl solution in methanol and incubated for 1.5h at 90°C. Samples were cooled down for 10 min before adding 1 mL of hexane and 1 mL of water. After phase separation, 600 μL of the hexane upper layer containing FAMEs was used for further analysis. A double internal standard spike was applied during the esterification process. Firstly, 1.6 mg of methyl pentadecanoate (C15:0) was added before the samples were heated at 90 °C. Then, 1.6 mg of methyl tridecanoate (C13:0) was added during the hexane extraction step. FAMEs were quantified by GC-FID using a Nukol column (Breuer et al., 2013; Teuling et al., 2017). The GC was calibrated using TraceCERT FAME standards purchased from Supelco.

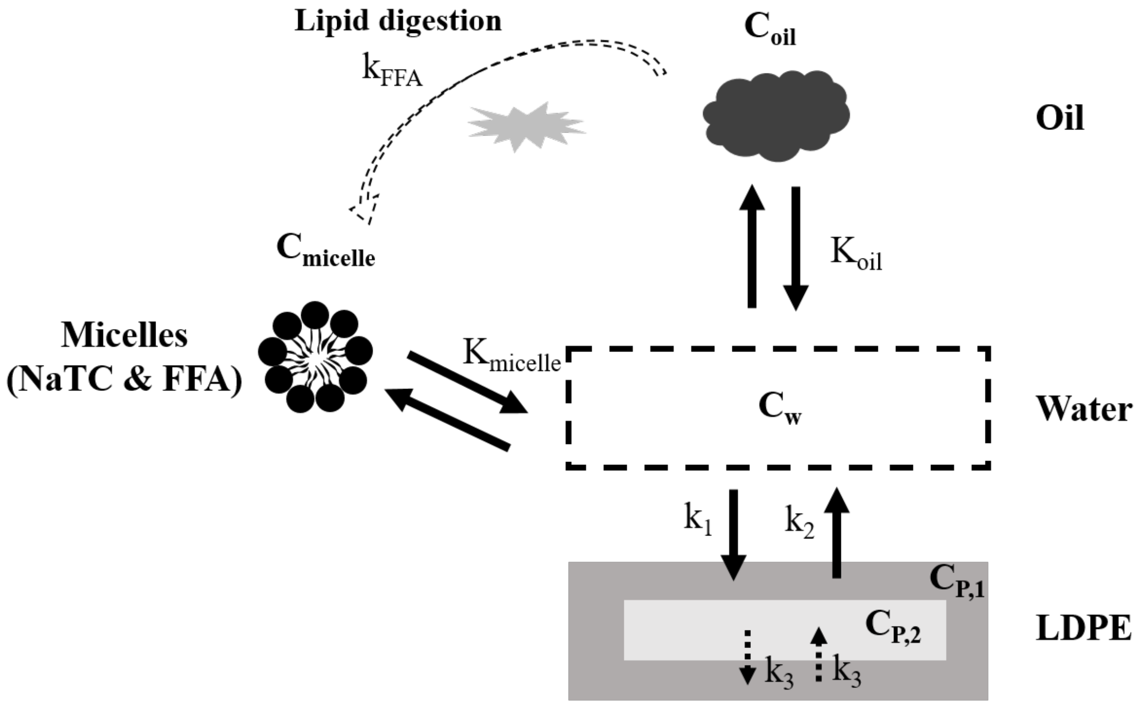

Figure S1. Schematic diagram of two-compartment low density polyethylene (LDPE) with a fast ($C_{P,1}$) and slow ($C_{P,2}$) reservoir and chemical interactions between LDP, water, oil and micelles (formed from sodium taurocholate (NaTC) and free fatty acids (FFA)) in the simulated gut fluid digestion assay.

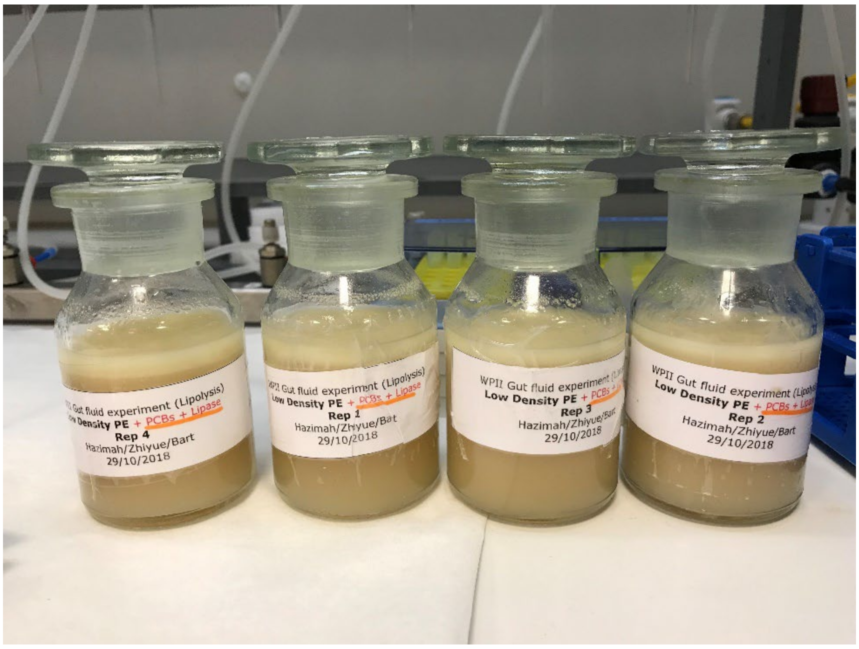

Figure S2. Picture of gut fluid set-ups

Determination of distribution coefficients $K_{micelle}$ and K_{oil}

According to chemical mass conservation, the mass of chemicals residing in the three phases of the system (i.e., POM, water and micelle/oil) remains equal to the initial mass of chemicals at all times. Here, we demonstrate the chemical mass balance for the micelle-water experiment:

$$M_{init} = M_{POM} + M_W + M_{micelle} \tag{S1}$$

where M_{init} is the total initial mass of PCB in the system, M_{POM} is the chemical mass on the POM passive sampler, M_W is the chemical mass in the water phase and $M_{micelle}$ is the chemical mass in the micelle compartment. All chemical mass are in µg. Each of the terms in eq. S1 are divided by the volume of the system and this yields:

$$C_{init} = C_{POM} + C_W + C_{micelle} \tag{S2}$$

Where each concentration term is in µg/L.

The partition equilibrium constant K_{POM} is:

$$K_{POM} = \frac{C_{POM}/[POM]}{C_W} \tag{S3}$$

where $[POM]$ is the mass concentration of POM in the system in kg/L (mass of POM divided by the total volume of the system) and K_{POM} is in L/kg. Hence,

$$C_{POM} = K_{POM} \cdot C_W \cdot [POM] \tag{S4}$$

Similarly, $C_{micelle}$ is:

$$C_{micelle} = K_{micelle} \cdot C_W \cdot [micelle] \tag{S5}$$

Where [micelle] is the mass concentration of micelle in the system in kg/L, $K_{micelle}$ (L/kg) is the partition coefficint of micelle with water.

Therefore, substituting eq. (S4) and (S5) into (S2):

$$C_{init} = K_{POM} \cdot C_W \cdot [POM] + C_W + K_{micelle} \cdot C_W \cdot [micelle] \tag{S6}$$

Rearranging and substituting $C_W = \frac{C_{POM}}{K_{POM}[POM]}$,

$$K_{micelle} = \frac{C_{init}K_{POM}[POM] - C_{POM}(K_{POM}[POM]+1)}{C_{POM}[micelle]} \tag{S7}$$

Similarly, for the oil-water partition coefficient experiment, the K_{oil} can be calculated from the following equation:

$$K_{oil} = \frac{C_{init}K_{POM}[POM] - C_{POM}(K_{POM}[POM]+1)}{C_{POM}[oil]} \tag{S8}$$

Biphasic sorption on microplastic model (based on Mohamed Nor and Koelmans, 2019)

The exchange of chemicals between the fast and slow reservoir of the plastic is modelled as:

$$\frac{dC_1{}^*}{dt} = \frac{k_1}{f_1[P]} C_W - k_2 C_1{}^* - k_3 C_1{}^* + k_3 C_2{}^* \quad \text{(S9)}$$

$$\frac{dC_2{}^*}{dt} = k_3 C_1{}^* - k_3 C_2{}^* \quad \text{(S10)}$$

where C_1^* and C_2^* are concentrations of contaminants in the fast and slow reservoirs of the polymer (µg/kg) respectively, f_1 is the fast reservoir fraction of the total bound mass of chemical (dimensionless), k_1 is the sorption rate constant (d^{-1}), k_2 is the desorption rate constant (d^{-1}) and k_3 is the intra-polymer rate constant (d^{-1}), [P] is the plastic mass concentration (kg/L).

Auxiliary equations

Relationship between log K_{POM} and log K_{OW} (Hawthorne et al., 2009)***:***

$$\log K_{POM} = 0.791 \cdot \log K_{OW} + 1.018 \quad \text{(S11)}$$

Percentage reduction in chemical bioavailability:

$$\%\, reduction\ in\ chemical\ bioavailability\ (t) =$$

$$\frac{(C_{tot}(0) - C_P{}^*(0) \cdot [P]) - (C_{tot}(t) - C_P{}^*(t) \cdot [P])}{(C_{tot}(0) - C_P{}^*(0) \cdot [P])} \times 100 \quad \text{(S12)}$$

Where $C_{tot}(0)$ and $C_{tot}(t)$ is the total concentration in the system (µg/L) at time 0h or t, $C_P{}^*(0)$ and $C_P^*(t)$ is the PCB concentration in LDPE (µg/kg) at time 0h or t, and [P] is the LDPE mass concentration in the system (kg/L).

Molecular weight of olive oil:

$$MW_{oil} = 3 \times Av.MW_{FFA} + 38.049 \quad \text{(S13)}$$

Where 38.049 g/mol is the the weight of the glycerol backbone and the average MW_{FFA} is 279.33 g/mol based on the percentage of fatty acids shown in Table S3.

Partition coeffcient of LDPE with water (Mohamed Nor and Koelmans, 2019)***:***

$$K_P = \frac{k_1}{k_2 f_1 [P]} \quad \text{(S14)}$$

For units, please refer to previous section.

Calculation of chemical contribution from small MP particles (<10 µm)

MP in the environment or in diet components relevant for human consumption typically follow a power law: 'Abundance= b*size^(-alpha)' with alpha (i.e., the power law parameter) typically having a value between 1.5 and 3 (average power law = 2.5) (Kooi et al., 2021). The average size of polydisperse particles 1 – 10 µm ($\mu_{x,poly}$) is calculated with (Kooi et al, 2021):

$$\mu_{x,poly} = \frac{1-\alpha_x}{2-\alpha_x} \times \frac{x_{UL}^{2-\alpha_x} - x_{LL}^{2-\alpha_x}}{x_{UL}^{1-\alpha_x} - x_{LL}^{1-\alpha_x}} \quad \text{(S15)}$$

In which α_x is the power law slope 'alpha' set at 2.5, x_{UL} is the upper limit of the range (here 10 µm) and x_{LL} is the lower limit of the default MP size range, set at 1 µm.

Equation S15 then yields an average size of MP particles in the range 1 to 10 µm, of 2.12 µm. The same calculation can be done for the other particles in a default MP size range i.e., from 10 to 5000 µm, which yields an average size of polydisperse particles between 10 and 5000 µm of 28.7 µm. Assuming uniform distribution of HOCs in the polymer, the chemical fraction present in the 1 – 10 µm particle fraction can be calculated from the relative particle volumes in these fractions, which scale to size with a power of three, i.e. fraction of chemical in the potentially translocatable fraction of particles is $2.12^3/(2.12^3 + 28.7^3) = 4\times10^{-4}$. This assumes all < 10 µm MP particles pass the gut lining, while this is actually around 0.3% (Mohamed Nor et al., 2021). This reduces the chemical fraction available for translocation to 4×10^{-7} of the total chemical mass present in ingested MP.

Given that the complementary fraction of particles that stays in the gut to be egested (i.e., $1 - 4\times10^{-7}$ = 0.9999996, or 99.99996 %), is subjected to the same 10 - 20 fold increase in chemical concentration, the net effect of MP mediated chemical transfer due to translocation will be overwhelmed (more than undone) by the attenuation of biomagnification and removal via egestion.

Note that using another alpha value, e.g. 2 or 3, does not affect this conclusion. Furthermore, extending the definition of MP to submicron particles (nanoplastics) reduces the average size of the particles < 10 µm even further, which further reduces the contribution via these small particles.

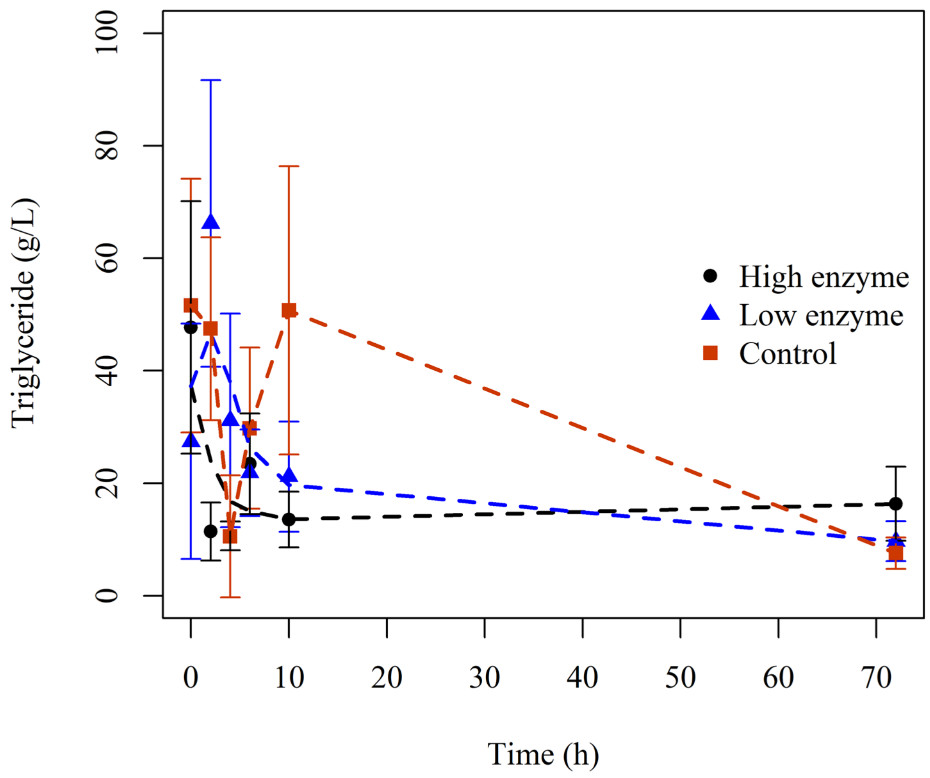

Figure S3. Concentration of triglycerides in the emulsion over time in each set-up (High enzyme, low enzyme and control)

Table S2. Average percentages of major fatty acids found in olive oil across samples.

Fatty acid	Average % of fatty acid	% Standard deviation
C14:0	0.03	0.02
C16:0	11.58	0.72
C18:0	2.68	0.15
C18:1	76.81	1.41
C18:2	7.69	0.42
C18:3	0.36	0.04
C20:0	0.65	0.09
C22:0	0.11	0.03
C22:1	0.02	0.02
C24:0	0.07	0.02

Table S3. Analysis of covariance (ANCOVA) to analyze the influence of different levels of micelle and oil mass concentrations on the partition coefficients, $K_{micelle}$ and K_{oil}, respectively over the log K_{OW} (covariate) range using the Type III test. ANCOVA to analyze the differences in the relationship of $logK_{micelle}$ vs $logK_{ow}$ from our study and Schacht et al. (2016). (Note: Covariates and treatments are independent. Variances among treatment groups are homogenous using Levene's test).

	Sum Sq.	Df	F value	Pr (>F)
$K_{micelle}$				
(Intercept)	0.29	1	6.87	0.0098 **
Log_Kow	102.81	1	2406.30	<2.2e-16***
Mass concentration level	10.49	3	81.86	<2.2e-16***
Residuals	5.94	139		
K_{oil}				
(Intercept)	10.60	1	162.33	<2.2e-16***
Log_Kow	57.70	1	883.30	<2.2e-16***
Mass concentration level	3.87	2	29.60	<2.2e-16***
Residuals	6.79	104		
Schacht et al. (2016)				
(Intercept)	1.19	1	10.57	0.00142**
Log_Kow	105.78	1	935.59	<2.2e-16***
Dataset	13.434	1	118.82	<2.2e-16***
Residuals	16.85	149		

*** $p < 0.001$; ** $p < 0.01$; * $p < 0.05$

Table S4. Post-hoc multiple pairwise comparisons using Tukey technique for $K_{micelle}$.

Levels	Estimate	Std. Error	t value	Pr (>|t|)
6.9g/L – 5.7g/L	-0.009	0.049	-0.184	0.998
11.6g/L – 5.7g/L	0.381	0.049	7.822	<1e-05***
64.5 g/L – 5.7g/L	0.630	0.049	12.940	<1e-05***
11.6g/L – 6.9g/L	0.390	0.049	8.006	<1e-05***
64.5g/L – 6.9g/L	0.639	0.049	13.124	<1e-05***
64.5g/L – 11.6g/L	0.249	0.049	5.118	<1e-05***

*** $p < 0.001$; ** $p < 0.01$; * $p < 0.05$

Table S5. Linear regressions between log $K_{micelle}$ and $logK_{OW}$ for different concentrations of micelles.

Experiment	Treatment	Regression	R^2
Micelle	5.7 g/L (Only NaTC)	$logK_{micelle} = 0.88 \cdot logK_{OW} + 1.09$	0.94
Micelle	6.9 g/L	$logK_{micelle} = 0.97 \cdot logK_{OW} + 0.44$	0.91
Micelle	11.6 g/L	$logK_{micelle} = 1.05 \cdot logK_{OW} + 0.36$	0.97
Micelle	64.5 g/L	$logK_{micelle} = 1.06 \cdot logK_{OW} + 0.54$	0.97
Micelle	5.7, 6.9, 11.6 g/L	$logK_{micelle} = 0.97 \cdot logK_{OW} + 0.63$	0.89
Micelle	All levels	$logK_{micelle} = 0.99 \cdot logK_{OW} + 0.61$	0.86

Table S6. Post-hoc multiple pairwise comparisons using Tukey technique for K_{oil}.

Levels	Estimate	Std. Error	t value	Pr (>|t|)
6.3g/L – 1.3g/L	-0.345	0.0602	-5.732	<1e-4
12.6g/L – 1.3g/L	-0.440	0.0602	-7.311	<1e-4
12.6g/L – 6.3g/L	-0.095	0.0602	-1.578	0.26

*** p < 0.001; ** p < 0.01; * p < 0.05

Table S7. Linear regressions between log K_{oil} and logK_{OW} for different concentrations of oil.

Experiment	Treatment	Regression	R^2
Micelle	1.3 g/L	$logK_{oil} = 0.85 \cdot logK_{OW} + 2.53$	0.89
Micelle	6.3 g/L	$logK_{oil} = 0.83 \cdot logK_{OW} + 2.27$	0.90
Micelle	12.6 g/L	$logK_{oil} = 0.89 \cdot logK_{OW} + 1.82$	0.90

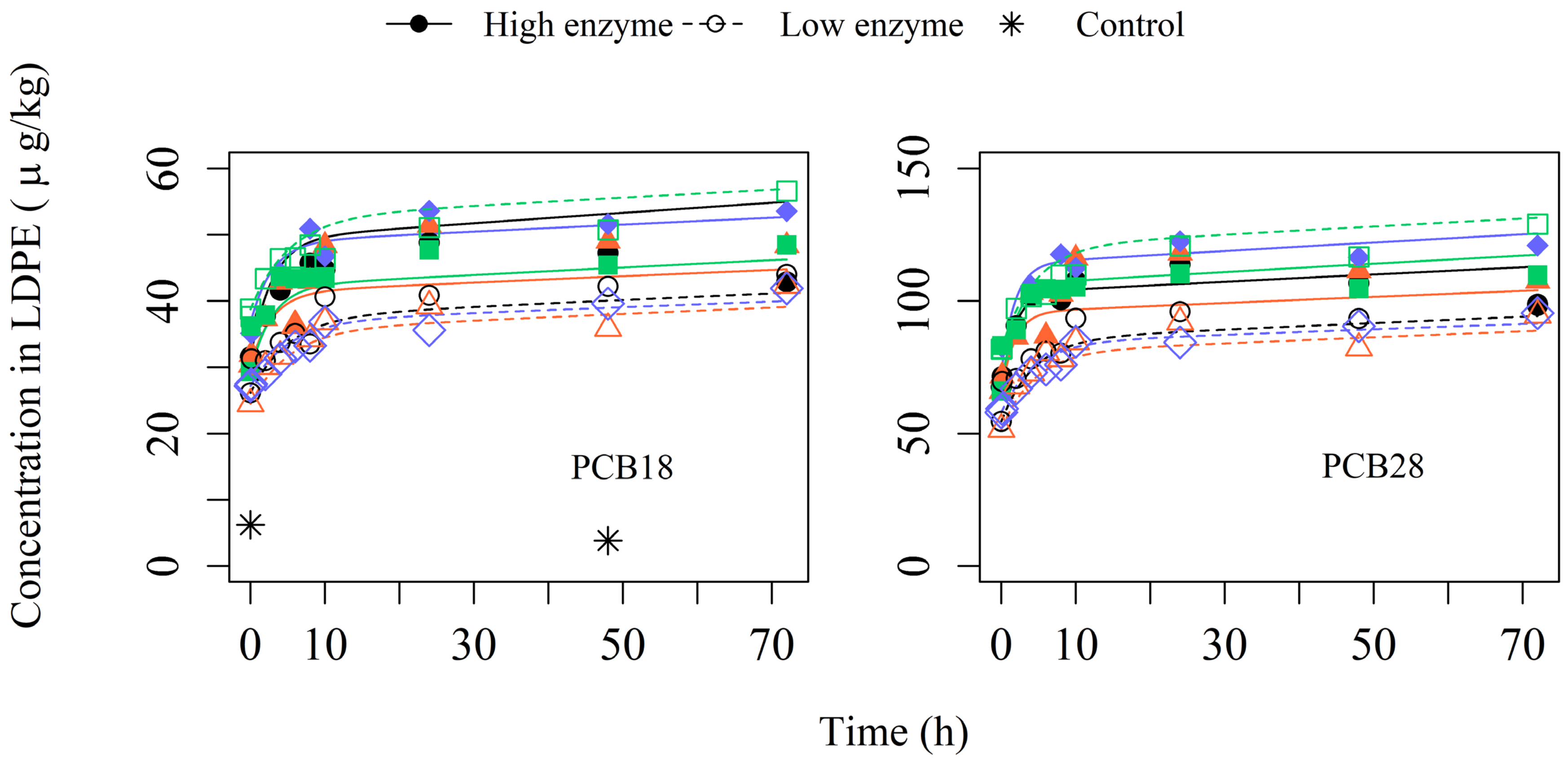

Figure S4. PCB concentrations in the LDPE compartment (µg/kg) over time (h) for high and low enzyme treatment and control (no PCBs spiked) of each replicate system. Solid lines represent the fitted models for the high enzyme treatment whereas the dashed lines represent the fitted models for the low enzyme treatment. Each colour represents a different replicate system.

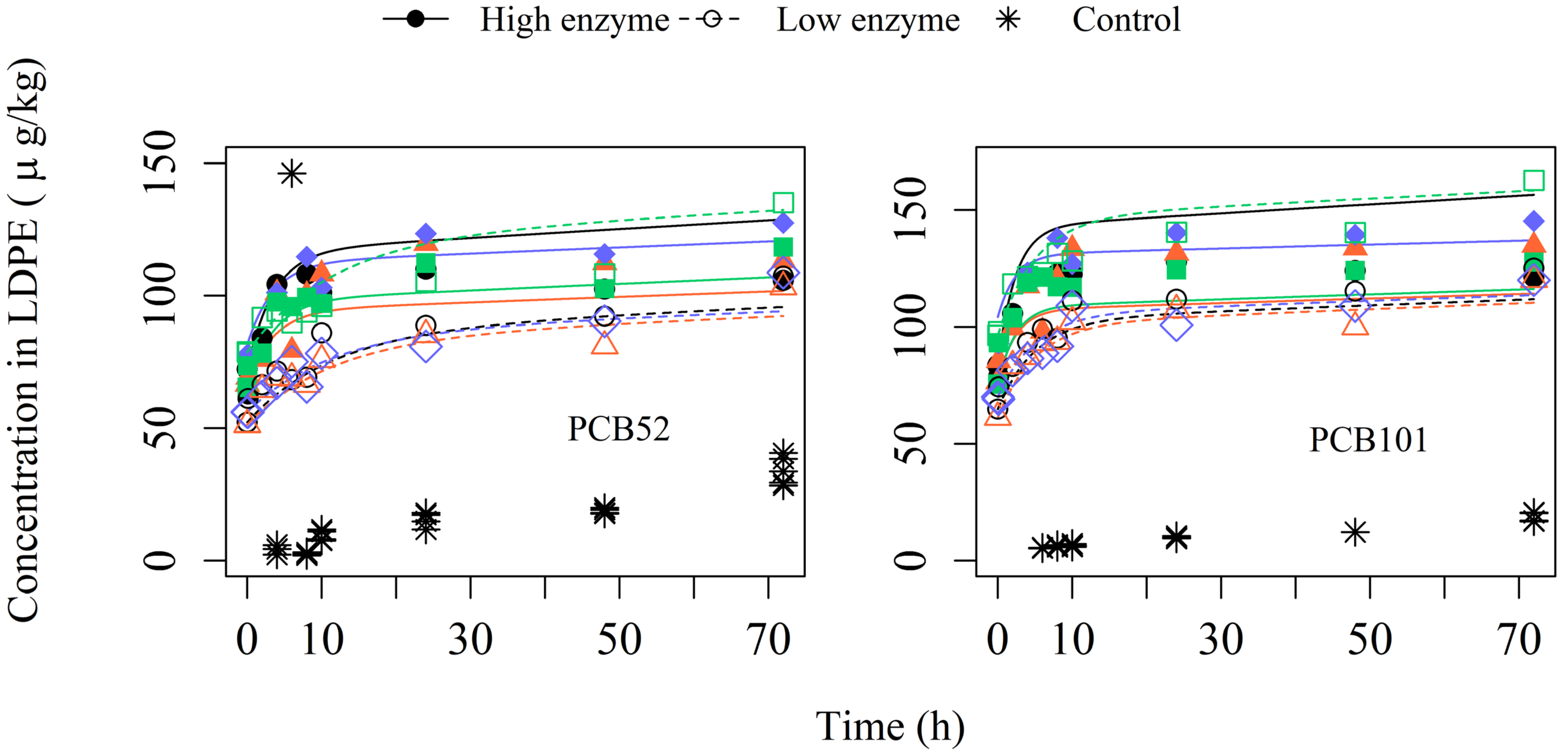

Figure S4. *(Continued)*

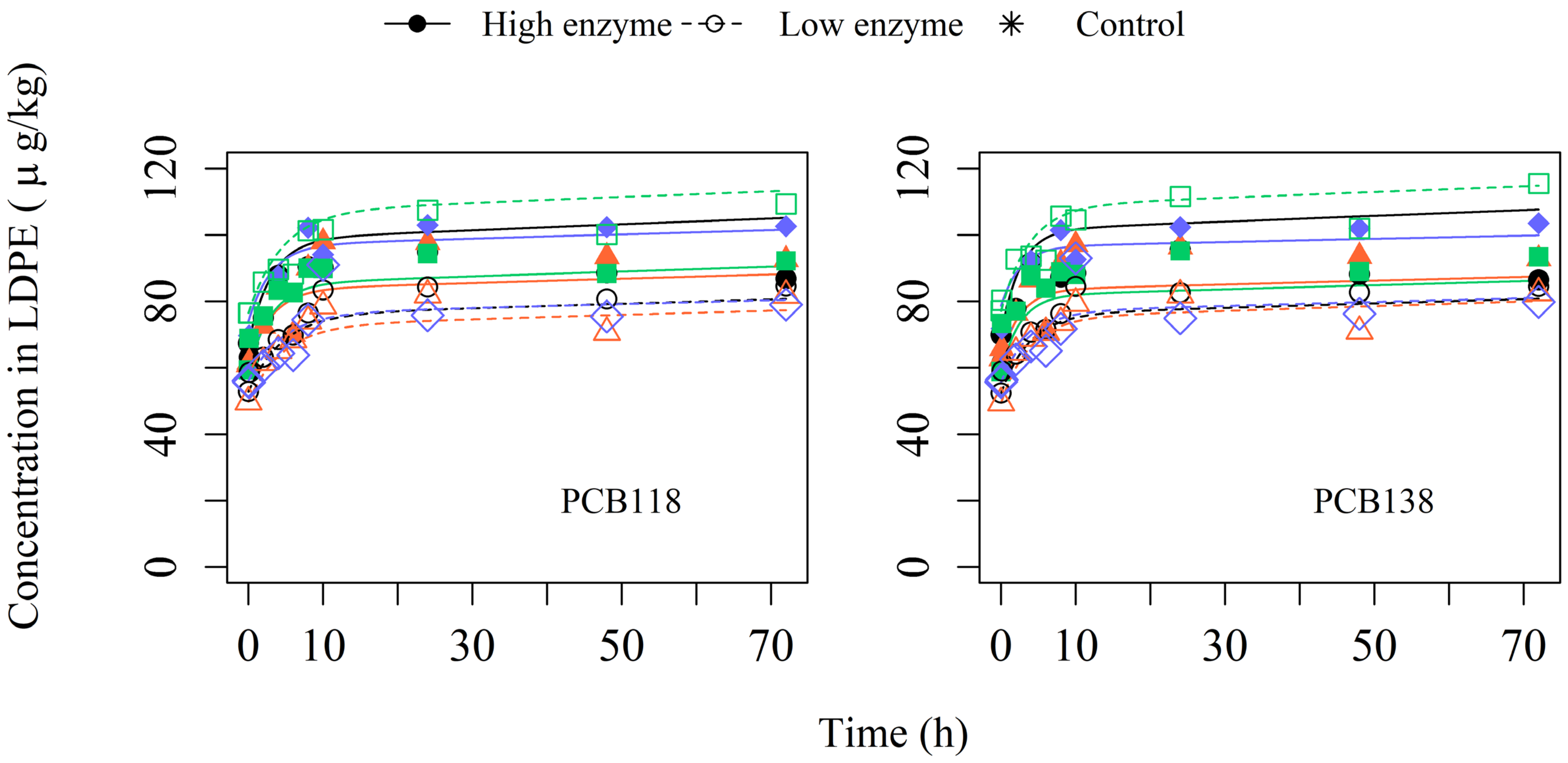

Figure S4. *(Continued)*

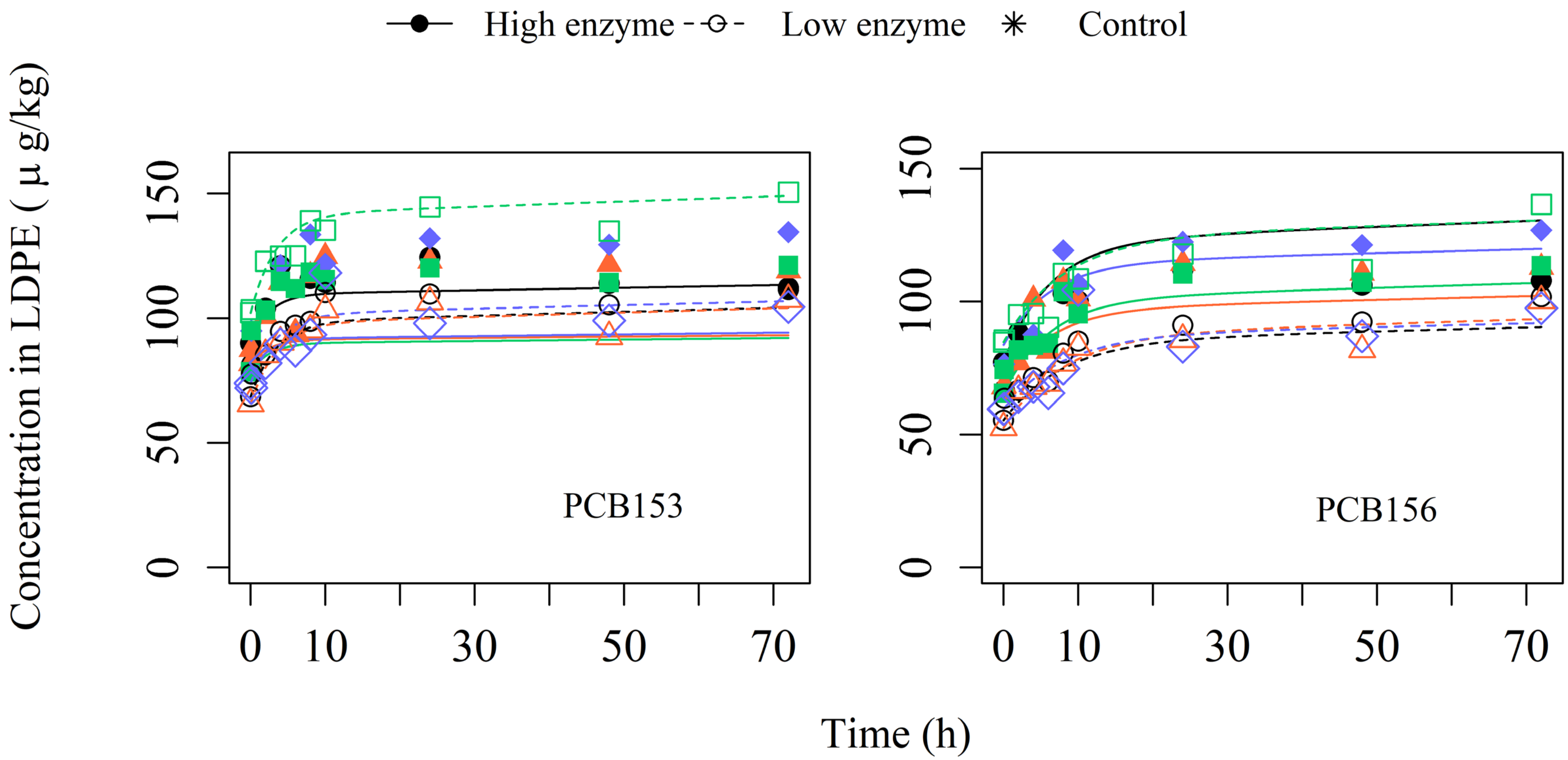

Figure S4. *(Continued)*

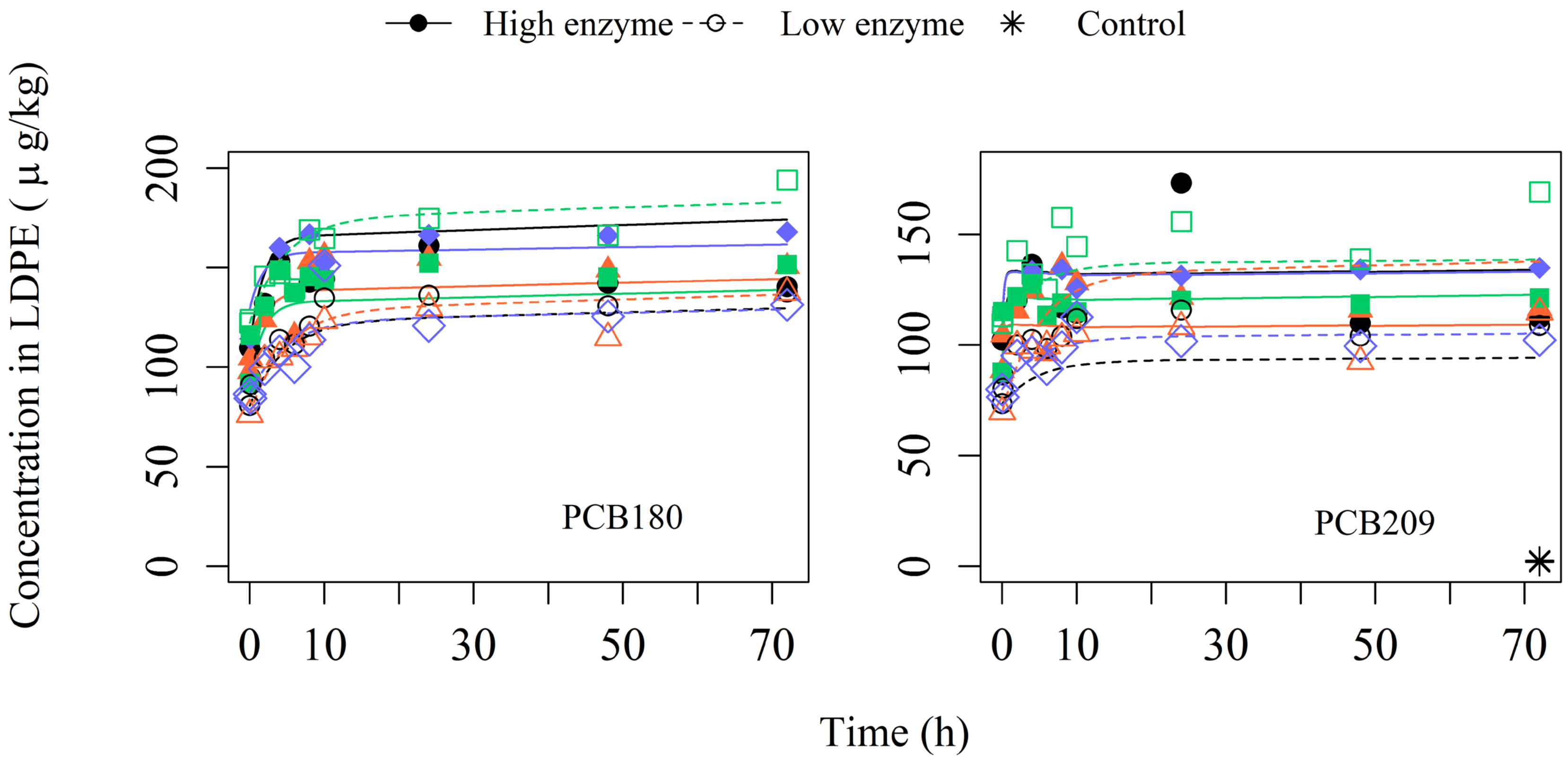

Figure S4. *(Continued)*

Table S8. Kinetic rate constants of LDPE for high and low enzyme treatment with standard errors (SE) (***significant at p<0.001; **significant at p<0.01; *significant at p<0.05)

Enzyme treatment	PCB congener	Log K_{OW}	k_1 (h^{-1})	SE	k_2 (h^{-1})	SE	K_P (L/kg)	log K_P
High	18	5.24	2.36E+02**	7.36E+01	0.345**	0.116	3.28E+05	5.52
High	28	5.67	1.61E+03**	4.50E+02	0.554**	0.163	1.21E+06	6.08
High	52	5.84	9.79E+02**	2.85E+02	0.283**	0.090	1.37E+06	6.14
High	101	6.38	5.59E+03*	2.39E+03	0.418*	0.190	4.44E+06	6.65
High	118	6.74	9.01E+03**	2.58E+03	0.322**	0.099	8.28E+06	6.92
High	138	6.83	1.40E+04*	5.88E+03	0.390*	0.173	1.03E+07	7.01
High	153	6.92	2.42E+04*	1.09E+04	0.477*	0.224	1.42E+07	7.15
High	156	7.18	1.33E+04***	3.38E+03	0.159**	0.045	2.15E+07	7.33
High	180	7.36	9.87E+04*	4.37E+04	0.550*	0.254	4.35E+07	7.64
High	209	8.18	1.37E+07	1.35E+07	4.355	4.316	5.85E+08	8.77
Low	18	5.24	1.38E+02***	2.09E+01	0.193***	0.033	3.42E+05	5.53
Low	28	5.67	6.04E+02***	8.40E+01	0.229***	0.036	1.10E+06	6.04
Low	52	5.84	3.33E+02***	5.43E+01	0.087***	0.017	1.51E+06	6.18
Low	101	6.38	3.13E+03***	4.15E+02	0.197***	0.030	5.28E+06	6.72
Low	118	6.74	5.92E+03***	1.08E+03	0.218***	0.044	8.04E+06	6.91
Low	138	6.83	1.00E+04***	1.77E+03	0.262***	0.051	1.10E+07	7.04
Low	153	6.92	1.58E+04***	2.81E+03	0.289***	0.056	1.53E+07	7.18
Low	156	7.18	1.03E+04***	1.75E+03	0.124***	0.024	2.13E+07	7.33
Low	180	7.36	4.22E+04***	7.04E+03	0.210***	0.039	4.87E+07	7.69
Low	209	8.18	6.32E+05**	2.14E+05	0.191*	0.071	6.16E+08	8.79

Table S9. Linear regression models of k_1 and k_2 with K_{OW} for high and low enzyme treatments

Treatment	Parameter	Relationship	R^2	*p*-value
High	k_1	$\log k_1 = 1.04\ (\pm 0.11) \times \log K_{OW} - 3.01(\pm 0.73)$	0.92	3.32e-5
Low	k_1	$\log k_1 = 1.21\ (\pm 0.08) \times \log K_{OW} - 4.28\ (\pm 0.51)$	0.96	2.73e-7
High	k_2	$\log k_2 = -0.28\ (\pm 0.57) \times \log K_{OW} - 0.28\ (\pm 0.57)$	-0.13	0.79
Low	k_2	$\log k_2 = 0.03(\pm 0.06) \times \log K_{OW} - 0.91\ (\pm 0.41)$	-0.10	0.65

Table S10. Analysis of covariance (ANCOVA) to analyze the influence of enzyme treatments on the transfer kinetic rate constants, k_1 and k_2, respectively over the log K_{OW} (covariate) range using the Type III test. (Note: Covariate and treatment are independent. Variance between treatment groups are homogenous using Levene's test).

	Sum Sq.	Df	F value	Pr (>F)
k_1 (uptake kinetic rate constant)				
(Intercept)	3.45	1	71.71	2.6E-07***
Log_Kow	14.70	1	305.65	7.5E-12***
Enzyme	0.25	1	5.19	3.7E-02*
Residuals	0.77	16		
k_2 (desorption kinetic rate constant)				
(Intercept)	0.063	1	2.27	0.151
Log_Kow	0.001	1	0.029	0.866
Enzyme	0.381	1	13.82	0.002**
Residuals	0.442	16		

*** p < 0.001; ** p < 0.01; * p < 0.05

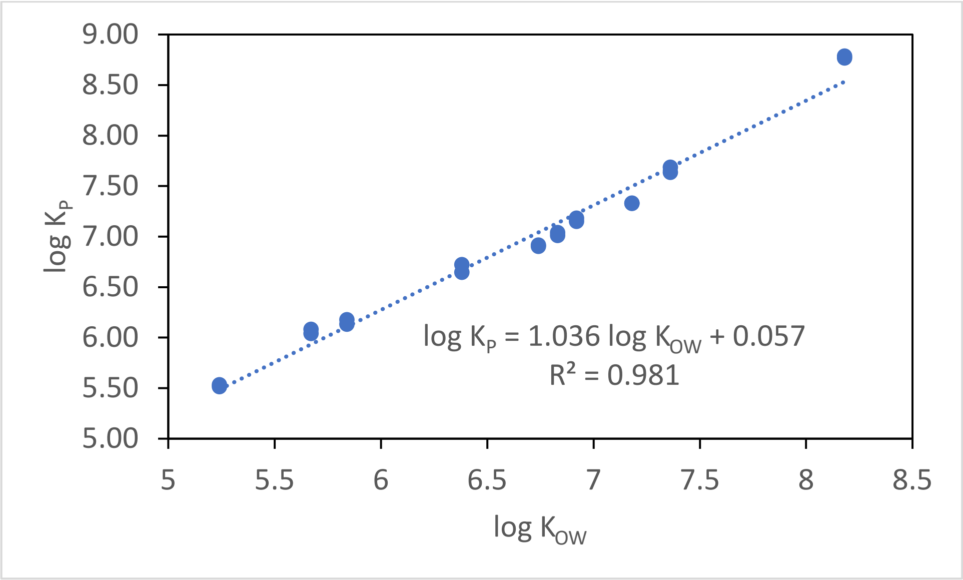

Figure S5. Relationship between log K_P of LDPE and log K_{OW} based on k_1 and k_2 estimates.

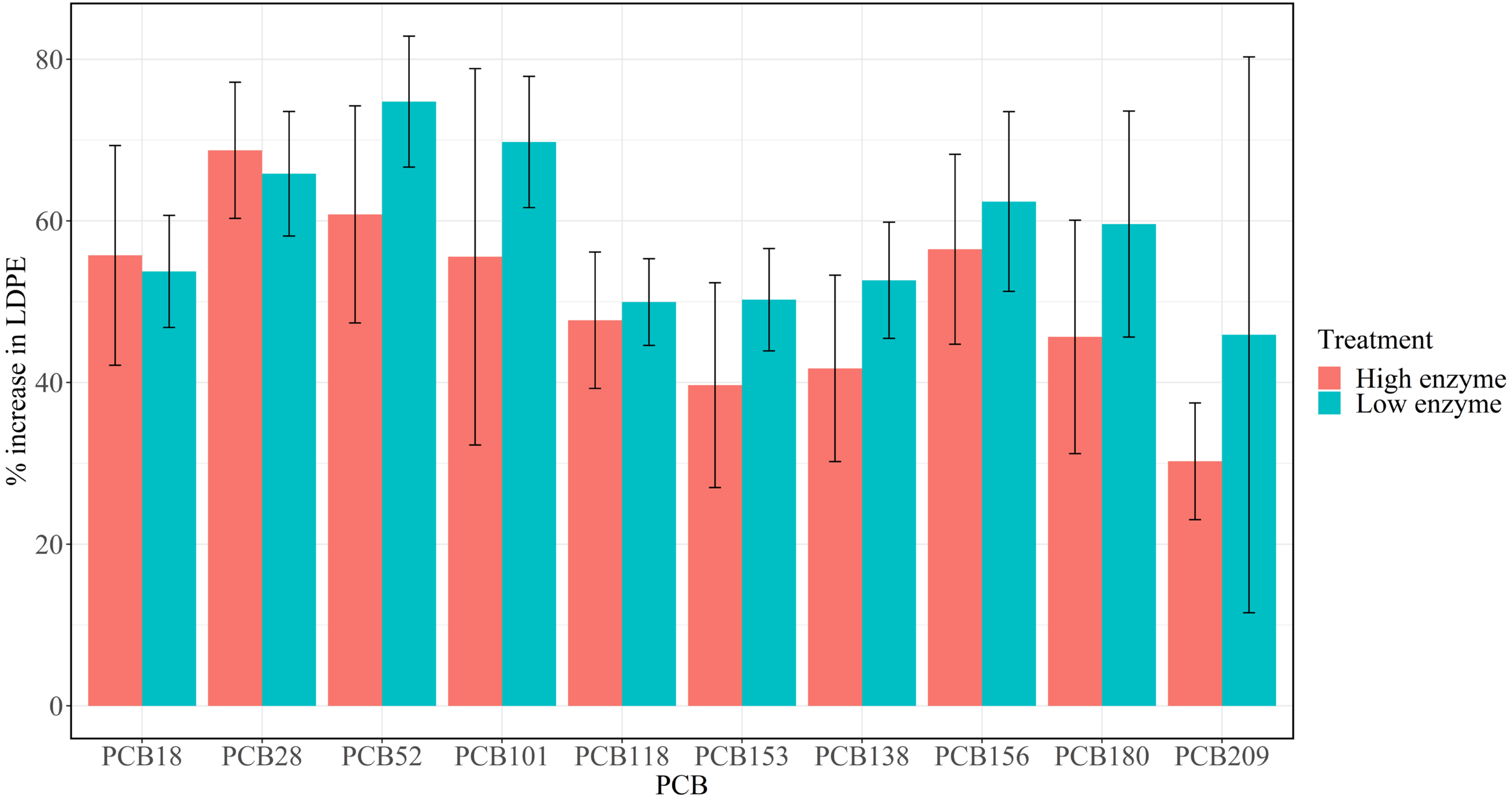

Figure S6. Percentage increase in LDPE after 72h of lipase digestion.

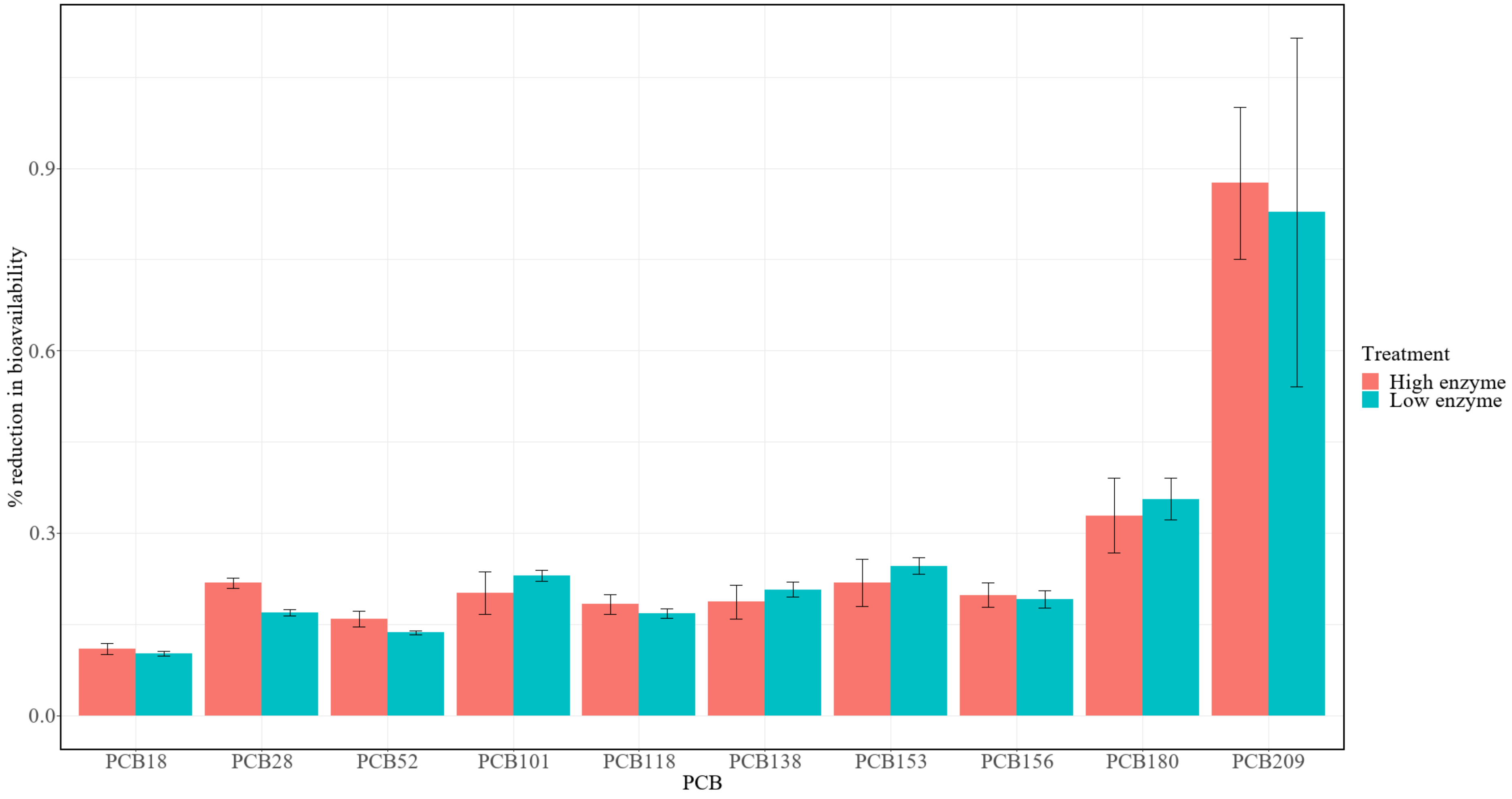

Figure S7. Percentage reduction in bioavailability of each PCB congener in high and low enzyme treatments respectively (based on empirical data: plastic is removed from system at every sampling timepoint)

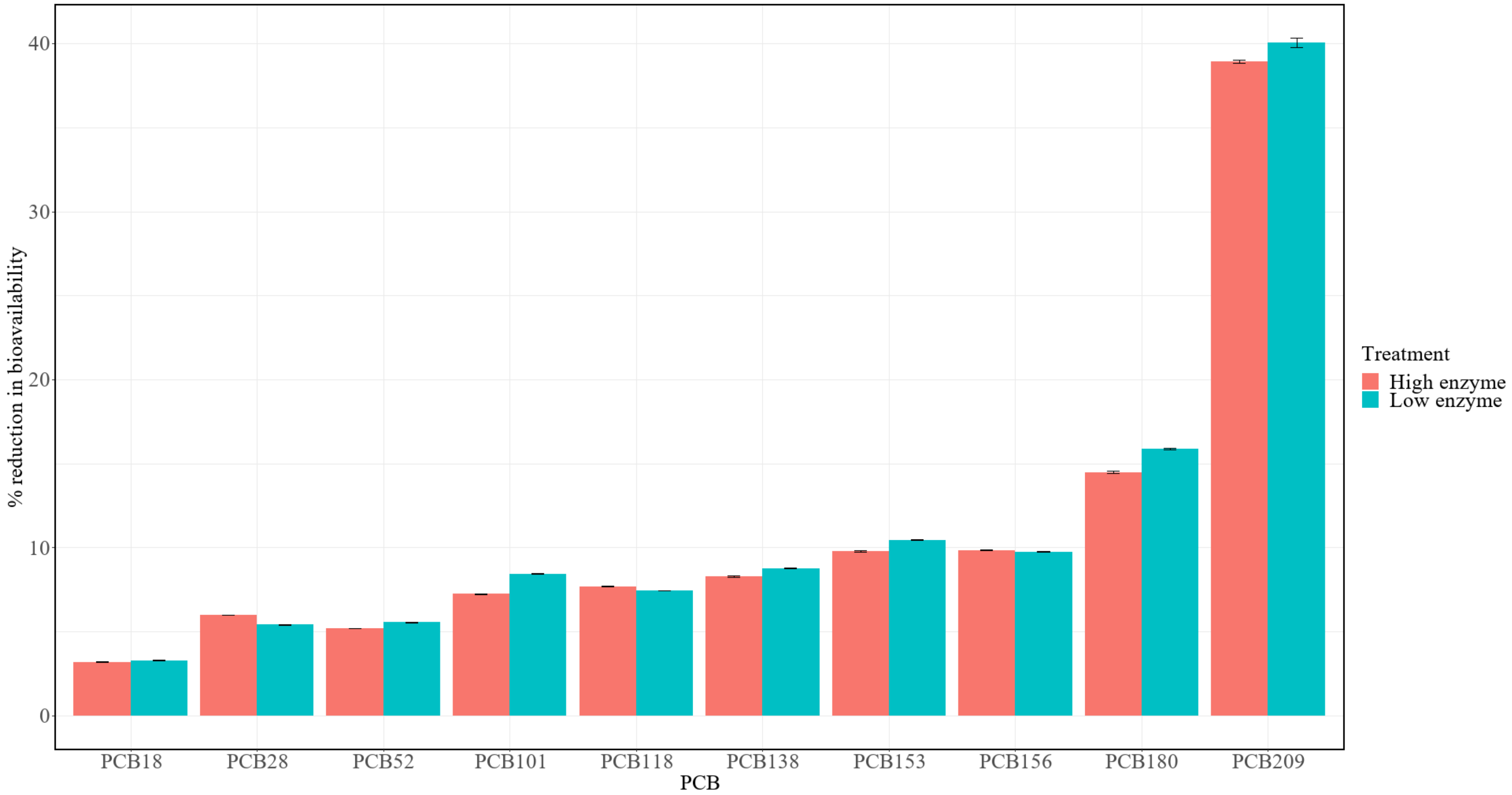

Figure S8. Percentage reduction in bioavailability of each PCB congener in high and low enzyme treatments respectively (based on simulated data: plastic remains constant in gut over time)

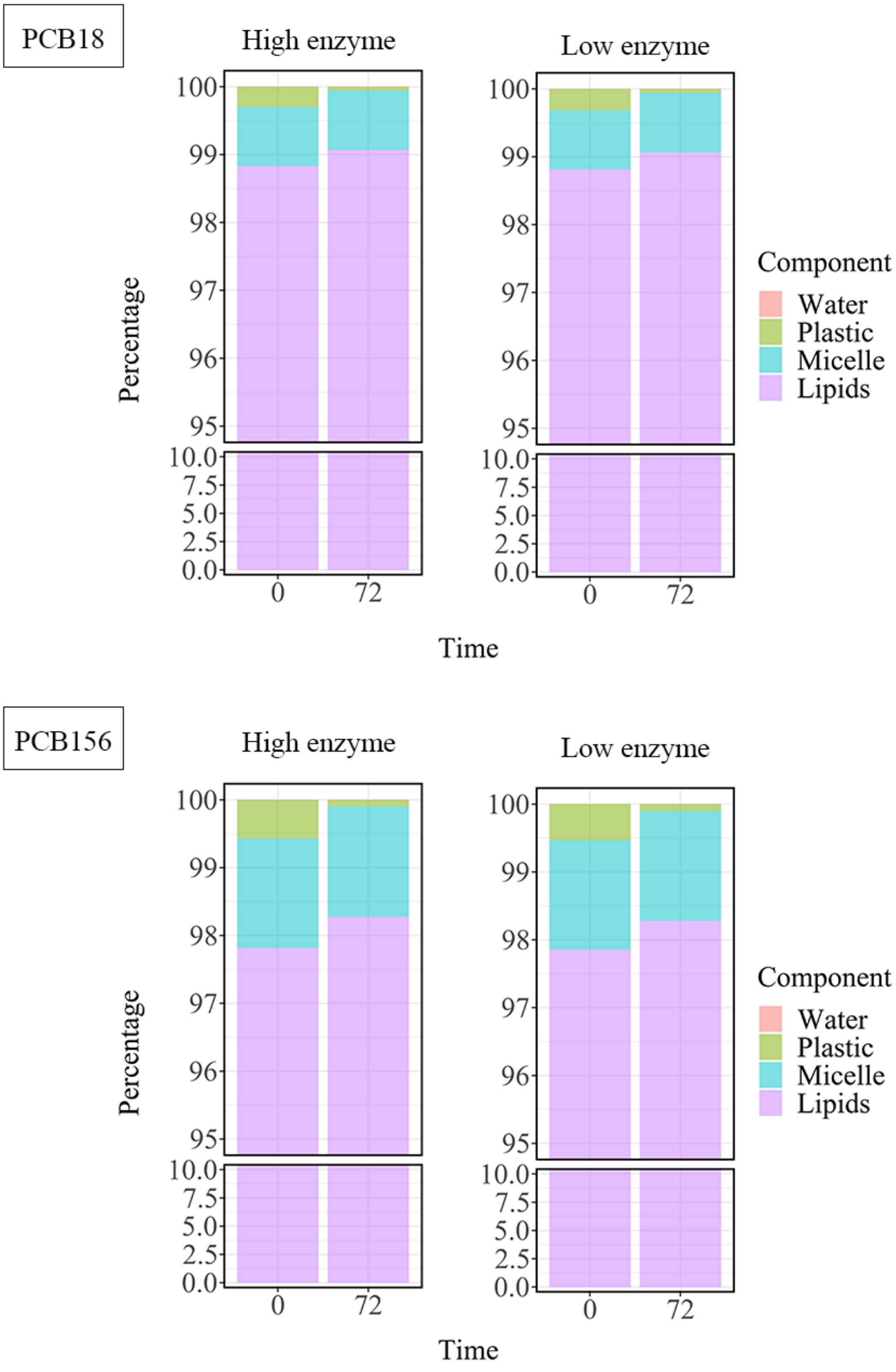

Figure S9. Percentage distribution of the PCB18 (top) and PCB156 (bottom) in each compartment of the system (i.e., water, plastic, micelle and lipids) at 0h and 72h after digestion for high enzyme (left panel) and low enzyme (right panel) treatment in the experiment.

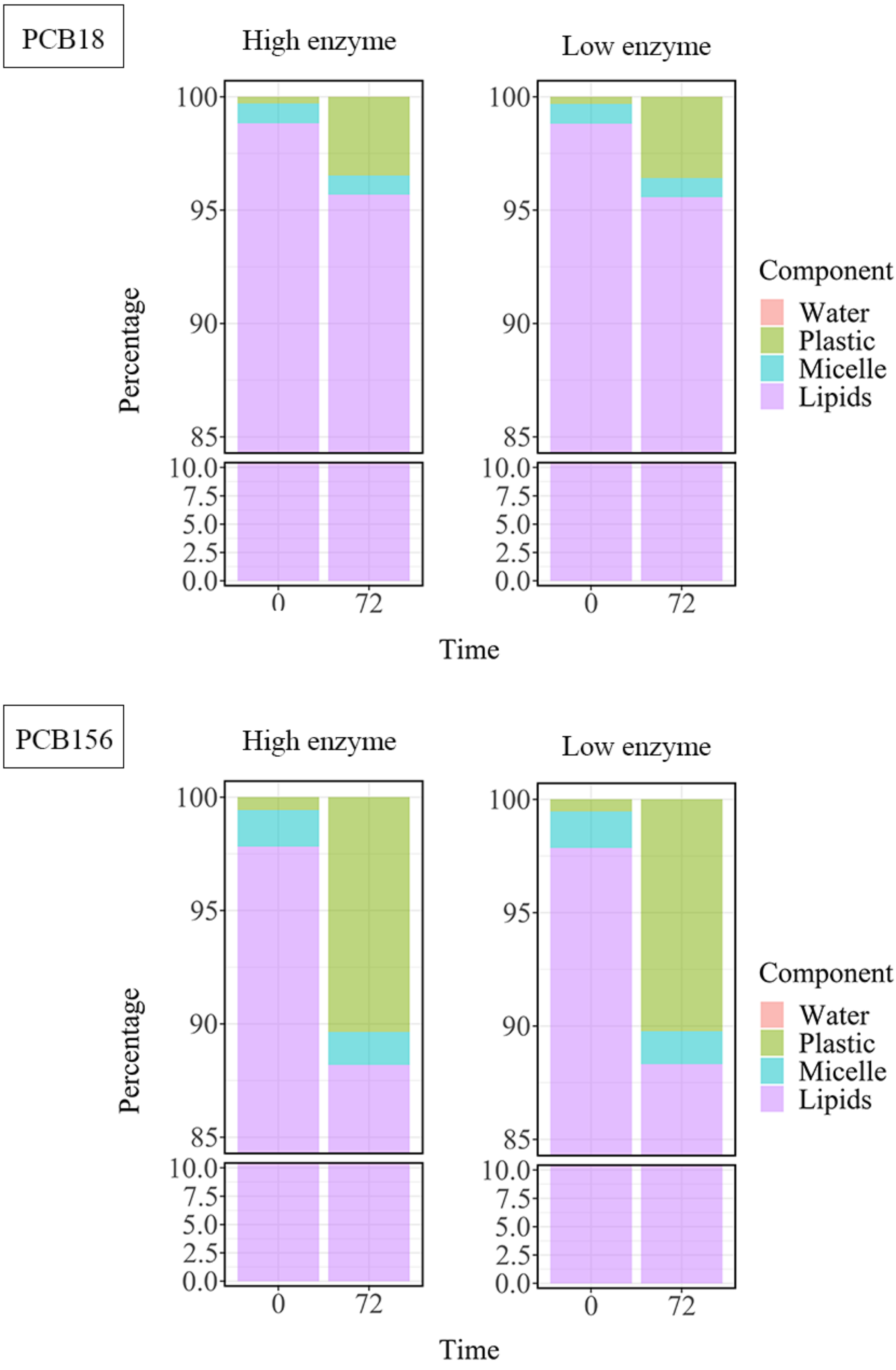

Figure S10. Percentage distribution of the PCB18 (top) and PCB156 (bottom) in each compartment of the system (i.e., water, plastic, micelle and lipids) at 0h and 72h after digestion for high enzyme (left panel) and low enzyme (right panel) treatment in the environmentally realistic scenario that the plastic remains constant over time.

References

Breuer, G., Evers, W.A.C., de Vree, J.H., Kleinegris, D.M.M., Martens, D.E., Wijffels, R.H., Lamers, P.P., 2013. Analysis of fatty acid content and composition in microalgae. J. Vis. Exp. 50628. https://doi.org/10.3791/50628

Hawker, D.W., Connell, D.W., 1988. Octanol-Water Partition Coefficients of Polychlorinated Biphenyl Congeners. Environ. Sci. Technol. 22, 382–387. https://doi.org/10.1021/es00169a004

Hawthorne, S.B., Miller, D.J., Grabanski, C.B., 2009. Measuring Low Picogram Per Liter Concentrations of Freely Dissolved Polychlorinated Biphenyls in Sediment Pore Water Using Passive Sampling with Polyoxymethylene. Anal. Chem. 81, 9472–9480. https://doi.org/10.1021/ac9019413

Kooi, M., Primpke, S., Mintenig, S. M., Lorenz, C., Gerdts, G., & Koelmans, A. A. (2021). Characterizing the multidimensionality of microplastics across environmental compartments. Water Research, 202, 117429.

Mohamed Nor, N.H.N.H., Koelmans, A.A.A.A., 2019. Transfer of PCBs from Microplastics under Simulated Gut Fluid Conditions Is Biphasic and Reversible. Environ. Sci. Technol. 53, 1874–1883. https://doi.org/10.1021/acs.est.8b05143

Mohamed Nor, N. H., Kooi, M., Diepens, N. J., & Koelmans, A. A. (2021). Lifetime accumulation of microplastic in children and adults. Environmental science & technology, 55(8), 5084-5096.

Paik, M.J., Kim, H., Lee, J., Brand, J., Kim, K.R., 2009. Separation of triacylglycerols and free fatty acids in microalgal lipids by solid-phase extraction for separate fatty acid profiling analysis by gas chromatography. J. Chromatogr. A. https://doi.org/10.1016/j.chroma.2009.06.051

Richardson, C.E., Hennebelle, M., Otoki, Y., Zamora, D., Yang, J., Hammock, B.D., Taha, A.Y., 2017. Lipidomic Analysis of Oxidized Fatty Acids in Plant and Algae Oils. J. Agric. Food Chem. https://doi.org/10.1021/acs.jafc.6b05559

Teuling, E., Wierenga, P.A., Schrama, J.W., Gruppen, H., 2017. Comparison of Protein Extracts from Various Unicellular Green Sources. J. Agric. Food Chem. 65, 7989–8002. https://doi.org/10.1021/acs.jafc.7b01788
